# Role of PATJ in stroke prognosis by modulating endothelial to mesenchymal transition through the Hippo/Notch/PI3K axis

**DOI:** 10.1038/s41420-024-01857-z

**Published:** 2024-02-17

**Authors:** Aina Medina-Dols, Guillem Cañellas, Toni Capó, Montse Solé, Marina Mola-Caminal, Natalia Cullell, Marina Jaume, Laura Nadal-Salas, Jaume Llinàs, Lluis Gómez, Silvia Tur, Carmen Jiménez, Rosa M. Díaz, Caty Carrera, Elena Muiño, Cristina Gallego-Fabrega, Carolina Soriano-Tárraga, Laura Ruiz-Guerra, Josep Pol-Fuster, Víctor Asensio, Josep Muncunill, Aarne Fleischer, Amanda Iglesias, Eva Giralt-Steinhauer, Uxue Lazcano, Isabel Fernández-Pérez, Joan Jiménez-Balado, Marina Gabriel-Salazar, Miguel Garcia-Gabilondo, Ting Lei, Nuria-Paz Torres-Aguila, Jara Cárcel-Márquez, Jerònia Lladó, Gabriel Olmos, Anna Rosell, Joan Montaner, Anna M. Planas, Raquel Rabionet, Mar Hernández-Guillamon, Jordi Jiménez-Conde, Israel Fernández-Cadenas, Cristòfol Vives-Bauzá

**Affiliations:** 1 Neurobiology Laboratory, Research Unit, Hospital Universitari Son Espases, Health Research Institute of Balearic Islands (IdISBa), Palma, Spain; 2 Department of Biology, University of Balearic Islands (UIB), Institut Universitari d’Investigacions en Ciències de la Salut (IUNICS), Palma, Spain; 3 Neurovascular Research Laboratory, Vall d’Hebron Research Institute, Universitat Autònoma de Barcelona, Barcelona, Spain; 4 Neurology, Hospital del Mar Medical Research Institute, Barcelona, Spain; 5 Unit of Medical Epidemiology, Department of Surgical Sciences, Uppsala University, Uppsala, Sweden; 6 Neurology, Hospital Universitari Mútua de Terrassa/Fundacio Docència i Recerca Mútua Terrassa, Terrassa, Spain; 7 Stroke Pharmacogenomics and Genetics, Institut de Recerca Sant Pau (IR SANT PAU), Barcelona, Spain; 8 Department of Neurology, Hospital Universitari Son Espases (HUSE), Palma, Spain; 9 Department of Genetics (GEN-IB), HUSE, IdISBa, Palma, Spain; 10 Genomic & Bioinformatics Platform, IdISBa, Palma, Spain; 11 Department of Respiratory Medicine,, Hospital Universitari Son Espases-IdISBa Palma, Spain; CIBERES, Instituto de Salud Carlos III, Madrid, Spain; 12 CIBER of Respiratory Diseases (CIBERES), Madrid, Spain; 13 Institute of Biomedicine of Seville, IBiS/Hospital Universitario Virgen del Rocío/CSIC/University of Seville & Department of Neurology, Hospital Universitario Virgen Macarena, Seville, Spain; 14 Department of Neuroscience and Experimental Therapeutics, Institut d’Investigacions Biomèdiques de Barcelona (IIBB)-Consejo Superior de Investigaciones Científicas (CSIC), Barcelona, Spain; 15 Area of Neuroscience, Institut d’Investigacions Biomèdiques August Pi i Sunyer (IDIBAPS), Barcelona, Spain; 16 Department of Genetics, Microbiology & Statistics, IBUB, University of Barcelona (UB), Barcelona, Spain; 17 Institut de Recerca Sant Joan de Déu, Esplugues de Llobregat, Barcelona, Spain; 18 Centro de Investigación Biomédica en Red de Enfermedades Raras (CIBERER), Instituto de Salud Carlos III, Madrid, Spain

**Keywords:** Mechanisms of disease, Blood-brain barrier, Epithelial-mesenchymal transition

## Abstract

Through GWAS studies we identified *PATJ* associated with functional outcome after ischemic stroke (IS). The aim of this study was to determine PATJ role in brain endothelial cells (ECs) in the context of stroke outcome. *PATJ* expression analyses in patient’s blood revealed that: (i) the risk allele of rs76221407 induces higher expression of *PATJ*, (ii) *PATJ* is downregulated 24 h after IS, and (iii) its expression is significantly lower in those patients with functional independence, measured at 3 months with the modified Rankin scale ((mRS) ≤2), compared to those patients with marked disability (mRS = 4–5). In mice brains, *PATJ* was also downregulated in the injured hemisphere at 48 h after ischemia. Oxygen-glucose deprivation and hypoxia-dependent of Hypoxia Inducible Factor-1α also caused PATJ depletion in ECs. To study the effects of *PATJ* downregulation, we generated *PATJ*-knockdown human microvascular ECs. Their transcriptomic profile evidenced a complex cell reprogramming involving Notch, TGF-ß, PI3K/Akt, and Hippo signaling that translates in morphological and functional changes compatible with endothelial to mesenchymal transition (EndMT). PATJ depletion caused loss of cell-cell adhesion, upregulation of metalloproteases, actin cytoskeleton remodeling, cytoplasmic accumulation of the signal transducer C-terminal transmembrane Mucin 1 (MUC1-C) and downregulation of Notch and Hippo signaling. The EndMT phenotype of PATJ-depleted cells was associated with the nuclear recruitment of MUC1-C, YAP/TAZ, β-catenin, and ZEB1. Our results suggest that *PATJ* downregulation 24 h after IS promotes EndMT, an initial step prior to secondary activation of a pro-angiogenic program. This effect is associated with functional independence suggesting that activation of EndMT shortly after stroke onset is beneficial for stroke recovery.

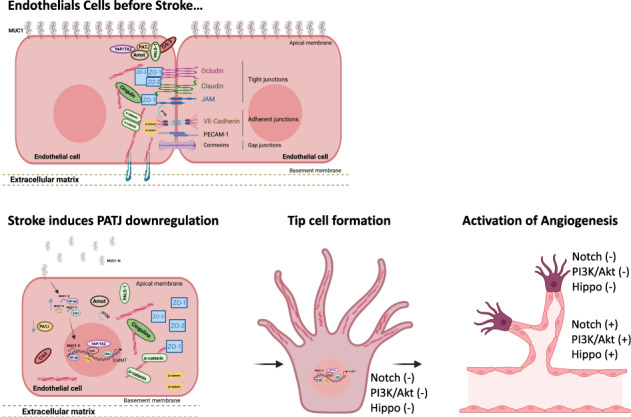

## Introduction

Stroke is a leading cause of long-term disability worldwide with substantial economic costs derived from patient care and treatment [1]. Hence, there is an urge for finding novel therapies to treat disability associated to stroke. This is the main reason why intensive research has focused on the discovery of key molecules involved in the functional prognosis of patients after stroke. By conducting a multicohort genome-wide association study (GWAS) metanalysis we identified *PATJ* low frequency variant rs76221407 (G allele) associated with worse functional outcome at three months after ischemic stroke (IS) [2]. PALS-1-associated tight junction protein (PATJ) is a scaffold protein constituent of the apicobasal polarity complex Crumbs, together with Crumbs3 (CRB3) and protein associated with LIN7 (PALS1) [3]. The Crumbs complex is localized in the most apical tip of the lateral membrane in epithelial cells, above the tight junctions (TJs) [4] and regulates epithelial polarity [5, 6], TJs formation and maintenance [7–9], apical domain identity and growth [10], cell migration [11, 12], actin organization and regulation of the Hippo pathway [3, 12–14]^,^. PATJ contains ten PDZ domains through which it mediates interaction with other proteins, as the components of the TJs *zonula occludens* 1 (ZO-1) and claudin-11 [15], or the proteins angiomotin (AMOT) [16, 17] and KIBRA [18], which link the Crumbs complex to the Hippo pathway [3].

The aims of this study were to disentangle the role of *PATJ* gene in stroke recovery, understanding how stroke affects *PATJ* expression depending on genetic variants, how this expression is related to stroke prognosis and which molecular mechanisms are involved in this process.

## Results

### *PATJ* downregulates after IS and lower expression levels associate with functional independence. The risk allele G of rs76221407 confers higher *PATJ* mRNA expression

**Fig. 1:**
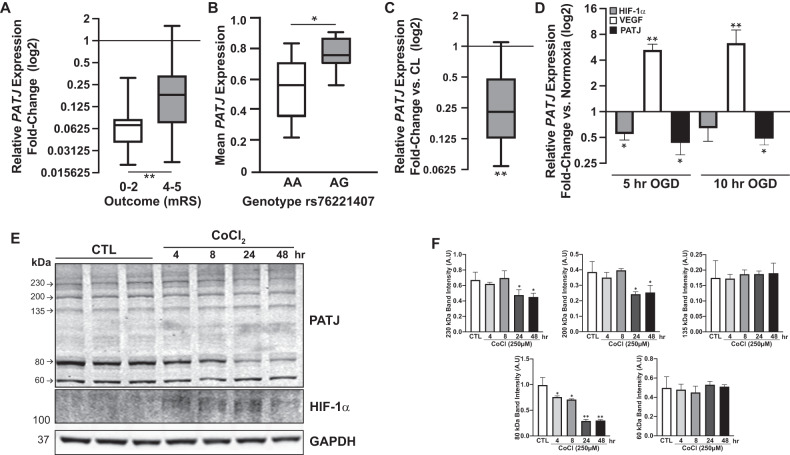
PATJ downregulation after IS associates with favorable functional outcome at 3 months. PATJ mRNA expression determined by RT-PCR (**A**, **C**, **D**) and by array-based gene expression (**B**). **A** Lower PATJ mRNA expression in peripheral blood extracted 24 h after IS in patients with functional independence (mRS < 3, *n* = 25) compared with patients with marked disability (mRS > 3, *n* = 25). *p < 0.05 as compared to mRS > 3 (Student’s t-test). **B** The G risk allele of rs76221407 SNP of *PATJ* gene confers higher expression in peripheral blood. *p < 0.05 as compared to the AA genotype (Student’s t-test). **C** *Patj* expression significantly decay in the ischemic region of mice brain subjected to 1 h transient middle cerebral artery occlusion. *Patj* mRNA levels were compared between the ipsilateral ischemic region (IL) and the contralateral CTL region (CL) of cortical brain homogenates, measured at 48 h after reperfusion. **p < 0.01 as compared to CL region (Paired t-test). **D** Oxygen-glucose deprivation (OGD) for 5 and 10 h induces *PATJ* downregulation in hCMEC/D3 ECs. **p < 0.01; *p < 0.05 as compared to normoxia (Student’s t-test). **E** PATJ steady-state levels significantly decrease in ECs subjected to HIF-1α-dependent chemical hypoxia. Depletion of the 80 kDa specie of PATJ observed after 4 h incubation with cobalt chloride (CoCl_2_). Prolonged incubation for 24 and 48 h accounted for depletion of the 230 and 200 kDa species. GAPDH was used as loading control. **F** Relative quantification of the integrated band intensities of three different WBs. Bars represent mean intensity ± SD. *p < 0.05; **p < 0.01 as compared to CTL cells (ANOVA followed by Bonferroni’s test).

We first determined in leukocytes how *PATJ* mRNA levels are modulated at 24 hours (h) after IS. 50 IS patients from the GODS cohort [2] were grouped depending on their 3-months mRS scores. Functional independent patients (mRS ≤ 0–2, (*n* = 25)) were compared with patients with marked disability (mRS ≥ 4, (*n* = 25)). *PATJ* mRNA levels decay after 24 h post-IS, but this depletion is significantly more pronounced on those patients with good functional outcome (Fig. 1A).

We next checked whether the risk allele G of SNP rs76221407 influence gene expression. For that purpose, we used the retrospective cohort GRECOS [19] of 77 healthy controls. Due to the low frequency of the SNP rs76221407, estimated to be 0.065 (1000Genomes), only five individuals presented heterozygosis (AG genotype), while the other 72 were homozygous (AA genotype). We found that the carriers of the G allele at rs76221407 had significantly higher expression of *PATJ* mRNA in blood (Fig. 1B).

*Patj* expression was also determined in brain homogenates from mice (*n* = 7) subjected to 1 h transient focal ischemia. 48 h after reperfusion, *Patj* mRNA levels significantly decreased by 4-fold in the ipsilateral ischemic region (IL) once compared to its contralateral control (CL) (Fig. 1C).

These results demonstrate that *PATJ* is downregulated both in blood and brain after IS and suggest that *PATJ* depletion in response to IS could be beneficial, since patients with protective mRS scores have lower *PATJ* expression levels. Moreover, individuals harboring the risk G allele at rs76221407 present higher *PATJ* expression.

### In human brain ECs PATJ depletion is mediated by the stabilization of hypoxia inducible factor-1α (HIF-1α)

To further characterize *PATJ* expression in response to hypoxia, human brain microvascular ECs (hCMEC/D3) were exposed to oxygen-glucose deprivation (OGD). OGD treatment significantly decreased *PATJ* expression by 2-fold after 5- and 10-h incubation (Fig. 1D). Vascular endothelial growth factor (VEGF), a target gene of HIF-1α, increased more than 4-fold, meanwhile HIF-1α mRNA levels decreased, as described elsewhere [20]. To explore whether *PATJ* expression is HIF-1α-dependent, ECs were subjected to chemical hypoxia by incubation with the hydroxylase inhibitor cobalt chloride (CoCl_2_, 150 µM) for 4, 8, 24, and 48 h. PATJ expression was studied by western-blot (WB). Five main PATJ species (230, 200, 135, 80, and 60 kDa) were identified in hCMEC/D3 cells using the PATJ antibody against the 800-1000 domain [21]. After 4- and 8-h of HIF-1α stabilization, the only PATJ species downregulated was the 80 kDa, but prolonged (24 and 48 h) CoCl_2_ incubation also translated in depletion of the PATJ higher molecular mass species of 230, and 200 kDa (Fig. 1E, F).

### *PATJ* knockdown (KD) causes loss of polarity and disruption of TJs in hCMEC/D3 cells

**Fig. 2:**
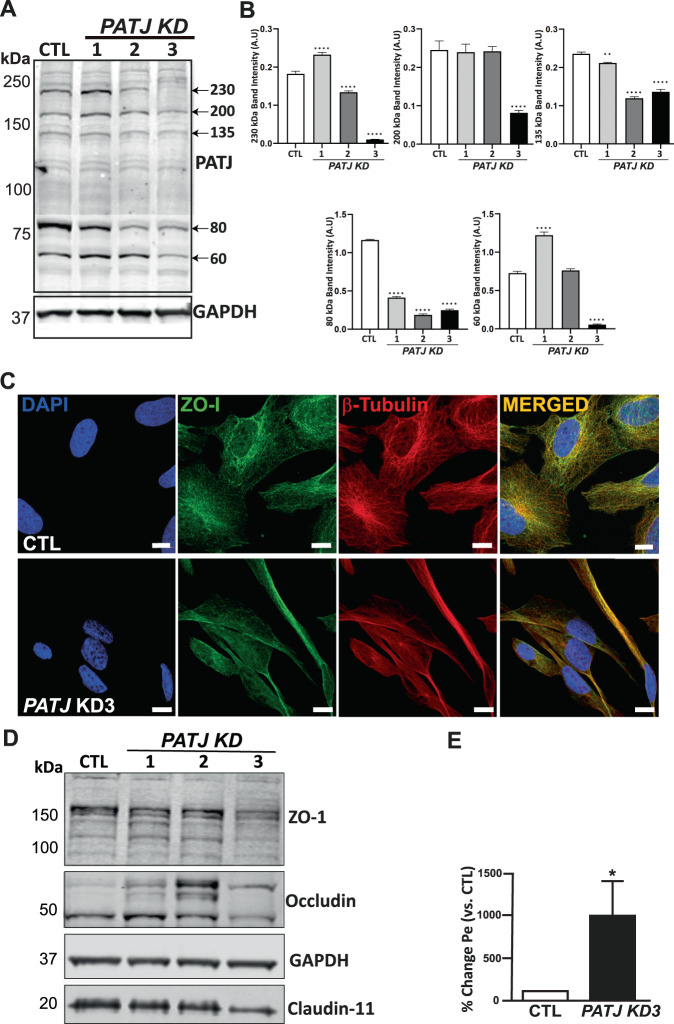
PATJ depletion in human brain microvascular endothelial cells (hCMEC/D3) causes the loss of tight junctions and confers a dramatic morphological change. **A** WB showing PATJ expression in three clones. Five main species were identified (230, 200, 135, 80, and 60 kDa). GAPDH was used as loading control. **B** Relative quantification of the integrated band intensities of three different WBs for PATJ species. Bars represent mean intensity ± SD. *p < 0.05; **p < 0.01; ***p < 0.005 and ****p < 0.0001 as compared to CTL cells (ANOVA followed by Bonferroni’s test). **C** *PATJ* KD3 cells lost their cell-cell interactions, as evidenced by immunofluorescence against the TJs protein *zonula occludens* 1 (ZO-1). **D** Differential expression of TJs proteins in the three *PATJ* KD cells. WB analyses showed that the loss of TJs in *PATJ* KD3 was due to the depletion of its constituent proteins ZO-1, occludin, and claudin-11. The two intermediate *PATJ* KD clones (KD1 and KD2) had different immunoreactive bands for occludin and ZO-1 than CTLs. **E** Transwell permeabilization assay evidenced a complete loss of permeability of the *PATJ* KD3 cells. *p < 0.05 as compared to CTLs (Student’s t-test).

To deeper study the molecular events underlying *PATJ* downregulation in response to IS, we generated hCMEC/D3 stably depleted of *PATJ* using lentiviral particles harboring shRNA against *PATJ*. Several clones were generated (Suppl. Figure 1A), three of which were selected for further analysis due to a progressive depletion of the PATJ species (Fig. 2A). *PATJ* KD1 clone only downregulated the 135 kDa (−10.2% ± 1.5) and the 80 kDa species (−64.4% ± 1), *PATJ* KD2 had depleted the 230 kDa (−17% ± 2), 135 kDa (−49.6% ± 3) and the 80 kDa (−83.9% ± 1.5) species, and *PATJ* KD3 was the only clone with depletion of all five PATJ species (230 kDa (−94.5% ± 2), 200 (−66.7% ± 3), 135 (−42.6% ± 4), 80 (−79.7 ± 0.7), and 60 kDa (−92.6% ± 1.6) (Fig. 2B).

Since PATJ contributes to TJs formation [5, 21–23], we first determined whether *PATJ* KD affected TJs in hCMEC/D3 cells. Immunofluorescence assay for the TJ marker zonula occludens-1 (ZO-1) revealed complete disappearance of ZO-1 staining at cell-cell contacts in *PATJ* KD3 clone (Fig. 2C). Loss of TJs in this clone correlated with downregulation of ZO-1, occludin, and claudin-11 (Fig. 2D). Interestingly, the two intermediate *PATJ* KD clones (KD1 and KD2) presented different immunoreactivity patterns than CTL cells for ZO-1 and occludin, with higher molecular species of occludin and lower species at ZO-1 (Fig. 2C). TJs disruption in *PATJ* KD3 cells was further confirmed with a permeability assay that exhibited one thousand times higher permeability to the Dextran tracer than CTL (Fig. 2E). Potentially higher sensibility of *PATJ* KD cells to tracer toxicity or reduced general cell survival due to PATJ depletion were discarded since EC viability, determined by MTT assay, demonstrated no differences between *PATJ* KD3 and CTLs (Suppl. Figure 1B).

### *PATJ* knockdown leads to downregulation of the Notch, PI3K/Akt, and Hippo pathways and translates into endothelial to mesenchymal transition

**Fig. 3:**
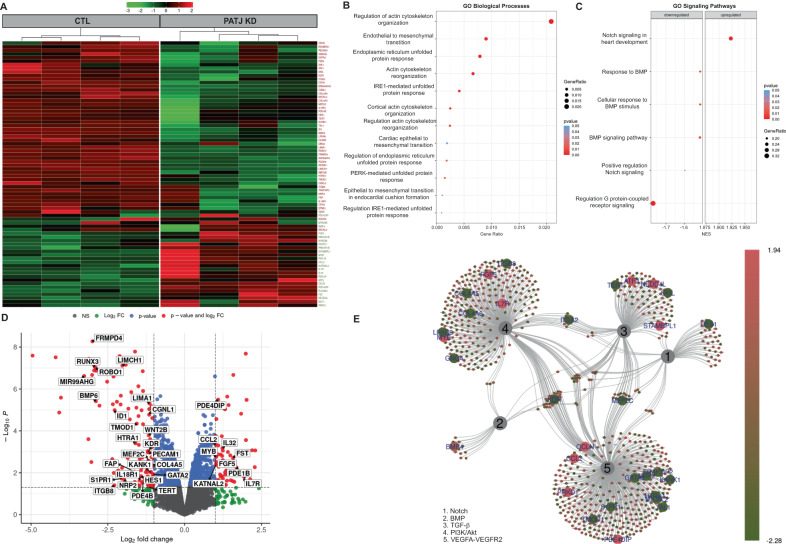
*PATJ* KD causes a massive transcriptomic reprogramming. **A** Hierarchical cluster showing the 74 most differentially expressed genes between *PATJ* KD and CTLs in the normalized (Log2 fold change (FC)) heatmap. *PATJ* KD1, 2, 3, and 4 were used. Level of expression was represented by color scale from green (low) to red (high), as indicated by the scale bar at the top. **B** Gene ontology (GO) analysis among the significantly modulated genes with an FDR cut-off of 0.05 revealed enrichment of biological processes such as Endothelial to Mesenchymal Transition (EndMT), Unfolded Protein Response (UPR) and Actin Cytoskeleton Organization. The results are visualized as a dot plot with an X-axis representing the gene ratio and the Y axis representing the FDR *p*-value. **C** Gene Set Enrichment Analysis (GSEA) for Gene Ontology (GO) and Kyoto Encyclopedia of Genes and Genomes (KEGG) enrichment between *PATJ* KD and CTL cells identified Notch, BMP and G-protein coupled receptor signaling as significant signaling pathways. **D** EnhancedVolcano plot of −log10 (*p*-values) vs. log2 FC. The −log10 (*p*-values) represents the level of significance of each gene while log2 FC represents the difference between the levels of expression for each gene between *PATJ* KD and CTL cells. **E** Gene-concept network (Cnetplot) showing the links between genes and the most significant cell signaling pathways, obtained with an FDR cut-off of 0.05, by using GSEA WikiPathways. The size of the concept nodes depends on the gene count involved in that pathway, while the color of the gene nodes depends on their expression level according to the displayed color gradient representing the log2 FC. Each number represents a significant pathway: NOTCH (1); BMP (2); TFG- β (3); PI3K/Akt (4) and VEGFA-VEGFR (5).

We noted that hCMEC/D3 cell morphology changed drastically due to *PATJ* KD, making the cells more elongated and spindle-like (Fig. 2C). This fact prompted us to hypothesize that *PATJ* depletion could induce a severe transcriptome reprogramming. Gene expression profiles, determined through the microarray platform, detected 341 differentially expressed genes identified through fold-change (FC) and P-value filtering adjusted using Bonferroni correction (FC ≥ 2 or FC ≤ −2 and P_adjusted_ < 0.05). Cluster analysis is shown in Fig. 3A. 137 genes were upregulated and 204 downregulated in *PATJ* KD cells compared with CTLs (Suppl. Table 1 and Suppl. Figure 2). Gene ontology (GO) analysis identified 124 categories that reached False Discovery Rate (FDR) significance (P_adjusted_ < 0.01) and highlighted as major modulated biological processes the actin cytoskeleton organization, endothelial to mesenchymal transition (EndMT) and the endoplasmic reticulum unfolded protein response (Fig. 3B and Suppl. Table 2). The major signaling pathways modulated in *PATJ* KD cells were Notch, Transforming Growth Factor-β (TGF-β)/Bone Morphogenic Proteins (BMP) signaling, PI3K/Akt, VEGFA-VEGFR2 signaling and Hippo (Fig. 3C, E and Suppl. Tables 2–3). These pathways are strongly related to each other, converging in the regulation of EndMT and angiogenesis (Fig. 3 and Suppl. Tables 2–3).

### *PATJ* KD cells express mesenchymal markers, switch off Notch, PI3K/Akt, and Hippo signaling

**Fig. 4:**
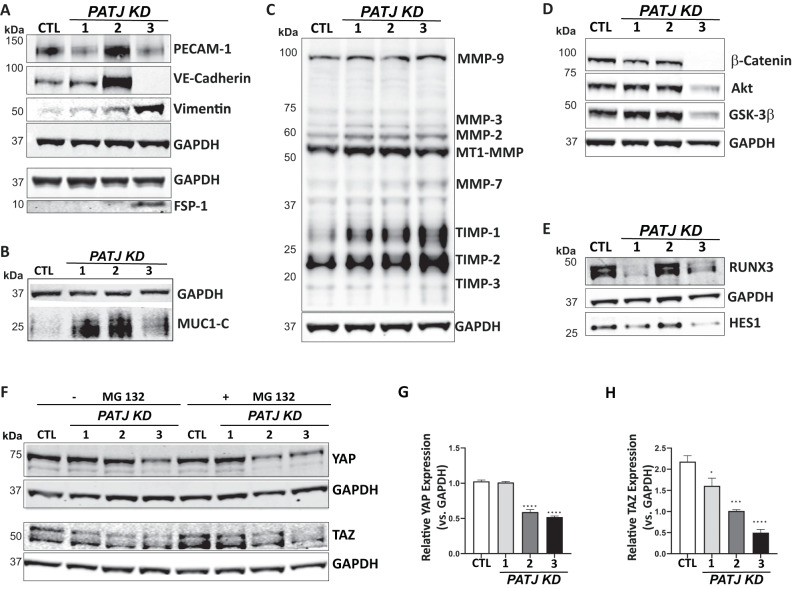
*PATJ* knockdown induces EndMT and downregulates the Notch, PI3K/Akt, and the Hippo pathways. WB analysis of: **A** EndMT markers PECAM-1/CD31, VE-Cadherin, Vimentin, and FSP-1/S100A4; **B** C-terminal subunit of mucin 1 (MUC1-C); **C** Matrix Metalloproteases (MMPs) and Tissue inhibitors of MMPs (TIMPs). **D** Notch signaling transcriptional modulators HES1 and RUNX3; **E** PI3K/Akt proteins Akt, GSK3-β, and β-catenin; **F** Hippo co-activators YAP/TAZ. GAPDH was used as loading control in all blots. **G**, **H** Relative quantification of the integrated band intensities of three independent WB for YAP (**G**) and TAZ (**H**). Bars represent mean intensity ± SD of three independent experiments. *p < 0.05; ***p < 0.005; ****p < 0.0001 as compared to CTL cells (ANOVA followed by Bonferroni’s test).

To validate the expression array data and to deeper investigate whether *PATJ* KD promotes EndMT and angiogenesis, protein markers [24, 25] were measured by WB. The endothelial marker VE-Cadherin was upregulated in the intermediates *PATJ* KD1 and KD2 clones and severely downregulated in *PATJ* KD3. The expression of PECAM-1/CD31, another endothelial marker, differed within the different *PATJ* KD clones, being downregulated in *PATJ* KD1 and *PATJ* KD3 and upregulated in *PATJ* KD2. Conversely, the mesenchymal marker Vimentin progressively upregulated in *PATJ* KD cells, inversely correlating with PATJ levels. Another mesenchymal marker, Fibroblast-specific protein 1 (FSP1/S100A4) was only expressed by *PATJ* KD3 (Fig. 4A). Loss of cell polarity has been associated with disruption of barrier epithelia and proteolytic cleavage of glycocalyx components as Mucin 1 (MUC1) [26]. Its cytoplasmic tail (MUC1-C) is a signal transducer involved in EndMT and hypoxia-driven angiogenesis [27, 28]. MUC1-C steady-state levels significantly increased in the three *PATJ* KD clones, with higher accumulation in the two intermediate *PATJ* KD1 and KD2 clones (Fig. 4B). Expression of matrix metalloproteinases (MMPs) is another major attribute that ECs acquire after undergoing EndMT [25, 29]. MMP-3, MMP-2, MT1-MMP and MMP-7 upregulated in *PATJ* KD clones, as well as the mesenchymal markers tissue inhibitors of MMPs, TIMP-1, 2, and 3 (Fig. 4C). These results suggest that the three *PATJ* KD clones analyzed were at different stages of EndMT, being *PATJ* KD1 and KD2 at the transient state, which is referred to “Partial EndMT” or “endothelial mesenchymal activation” (EndMA) [25, 29], and only the *PATJ* KD3 cells acquired a complete mesenchymal phenotype.

We next investigated the pathways involved in triggering EndMT-angiogenesis upon PATJ depletion. Both, Notch and PI3K/Akt signaling, central coordinators of EndMT-angiogenesis [30–33], were off in *PATJ* KD3 cells with the mesenchymal phenotype (Fig. 4D, E). By contrary, the transient state of the intermediate *PATJ* KD clones partially downregulated the PI3K/Akt, as evidenced with upregulation of GSK3β and downregulation of β-catenin (Fig. 4D), and differed in Notch signaling, being partially off in *PATJ* KD1, with depletion of the transcription factors RUNX3 and HES1, and maintained on in *PATJ* KD2 (Fig. 4E). These results suggest a modulatory role of PI3K/Akt and Notch pathways along the EndMT process induced by PATJ depletion, being partially active at the transient stages and switched off once the mesenchymal phenotype is achieved.

Hippo signaling, another pathway significantly modulated due to *PATJ* depletion, has also been described as a driver of EndMT-mediated angiogenesis [34, 35]. When the pathway is activated, its downstream transcriptional co-activators YAP (Yes-associated protein) and TAZ (Tafazzin) are phosphorylated and sequestered in the cytosol or degraded by the proteasome. When Hippo is inhibited, YAP/TAZ may enter the nucleus, in where activate the TEA domain family members (TEAD) transcription factors promoting gene expression, including genes involved in EndMT [35].

We first checked the steady-state levels of YAP/TAZ, incubating cells with or without the proteasome inhibitor MG132 (Fig. 4F). No differences in YAP levels were found in *PATJ* KD1, but *PATJ* KD2 and KD3 cells depleted YAP levels by almost 50% (Fig. 4F, G). Regarding TAZ, a progressive depletion was observed in the three *PATJ* KD clones, being more evident after MG132 treatment (Fig. 4F, H).

### *PATJ* KD cells recruit to the nucleus the signal transducer MUC1-C and the transcriptional modulators YAP/TAZ, β-catenin, and ZEB1

**Fig. 5:**
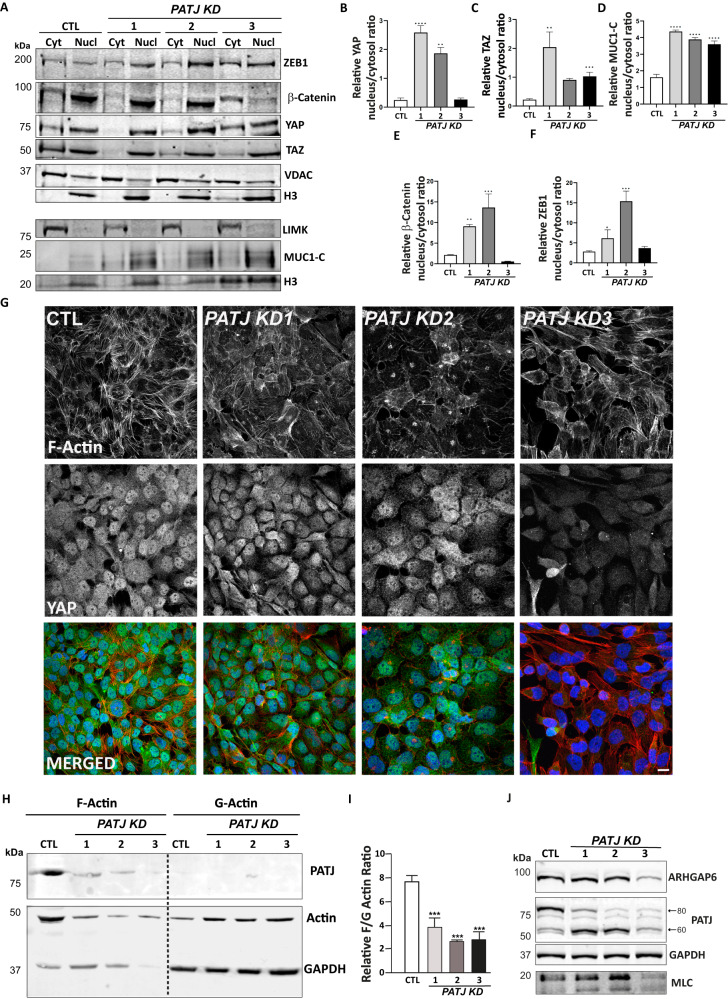
PATJ depletion causes nuclear translocation of YAP/TAZ, MUC1-C, β-catenin, and ZEB1 and actin cytoskeleton remodeling. **A** Cytosolic (Cyt) and Nuclear (Nucl) localization of ZEB1, β-catenin, YAP/TAZ, and MUC1-C. Voltage-dependent anion channel (VDAC) and LIM Kinase (LIMK) were used as cytosolic markers and Histone 3 (H3) as nuclear marker. **B**–**F** Quantifications of nuclear/cytosolic ratios for YAP (**B**), TAZ (**C**), MUC1-C (**D**), β-catenin (**E**) and ZEB1 (**F**). **G** Immunofluorescence using phalloidin to label F-actin (red) and YAP-antibody (green). **H**, **I** PATJ KD decreased the F/G actin ratio. **H** Representative immunoblot of F-actin and G-actin. **I** Relative quantification of the F/G actin ratio. Bars represent mean intensity ± SD of three independent experiments. ***p < 0.005 as compared to CTL cells (ANOVA followed by Bonferroni’s test). **J** WB analysis showing depletion of the actin cytoskeleton markers myosin light chain (MLC) and ARHGAP6 in *PATJ* KD3 cells.

We next evaluated whether YAP/TAZ could be translocated to the nucleus upon *PATJ* silencing. Intermediate *PATJ* KD clones had higher amounts of YAP in the nuclear fraction, contrarily to the low levels observed in *PATJ* KD3 cells (Fig. 5A, B). These results were also confirmed by YAP immunofluorescence (Fig. 5G). By contrary, TAZ was enriched in the nuclear fraction of all three *PATJ* KD clones (Fig. 5A, C).

Since MUC1-C has been shown to promote the nuclear translocation of YAP/β-catenin complexes [36], we also checked its subcellular distribution. Interestingly, MUC1-C was mostly recruited to the nucleus of *PATJ* KD clones (Fig. 5A, D). And as expected, the distribution pattern of β-catenin was like the one observed for YAP, being enriched in the nuclear fraction of the two intermediate *PATJ* KD clones (Fig. 5A, E). The transcriptional repressor ZEB1, a known EndMT inducer [37], which expression is controlled by MUC1-C [38], was also enriched in the nuclear fraction of the intermediate *PATJ* KD clones (Fig. 5A, F). These results suggest that the EndMA phenotype of intermediate *PATJ* KD clones is achieved by MUC1-C/YAP/TAZ/β-catenin/ZEB-nuclear localization. By contrary, cells that complete the mesenchymal transition, as *PATJ* KD3, only maintain nuclear MUC1-C/TAZ.

### PATJ depletion causes actin cytoskeleton remodeling

YAP/TAZ regulate actin remodeling at filopodia and cell-cell junctions [35], and actin cytoskeleton remodeling is necessary for EndMT and angiogenesis [39]. Moreover, “regulation of actin cytoskeleton organization” was the most significant GO biological process associated with *PATJ* knockdown (Fig. 3B). Therefore, we investigated the actin cytoskeleton dynamics in the *PATJ* KD clones. Immunofluorescence analysis using labeled phalloidin evidenced loss of stress fibers, but increased membrane ruffles and lamellipodia in the *PATJ* KD cells, although the two intermediate *PATJ* KD clones (KD1 and KD2) tended to maintain the cortical endothelial organization of the actin filaments (Fig. 5G). Loss of filamentous actin (F-actin) and concurrent increase in globular (G-actin) was further demonstrated by quantitative WB analysis in the three *PATJ* KD clones (Fig. 5H, I). Interestingly, key regulatory proteins of the actin cytoskeleton as the Rho A inhibitor Rho GTPase Activating Protein 6 (ARHGAP6) and Myosin Light Chain (MLC) were severely downregulated only in *PATJ* KD3 cells (Fig. 5J), suggesting a complex pattern of actin dynamics regulation.

### PATJ depletion induces endothelial activation and vascular inflammation through nuclear recruitment of MUC1-C and YAP

**Fig. 6:**
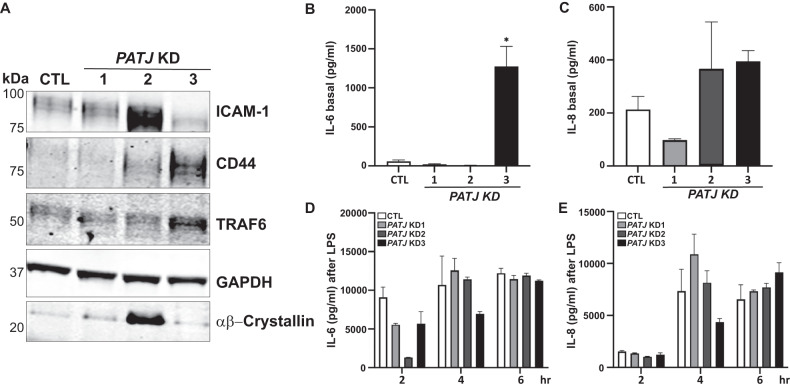
PATJ depletion induces a pro-inflammatory phenotype characterized by increased expression of cell adhesion molecules and production of IL-6 and IL-8. **A** Differential expression of the cell adhesion molecules ICAM-1 and CD44, as well as TRAF6 in *PATJ* KD cells, revealed by WB analysis. **B**, **C** Basal secretion of IL-6 (**B**) and IL-8 (**C**). *PATJ* KD3 cells produce significantly higher amounts of IL6. **D**, **E** Secretion of IL-6 (**D**) and IL-8 (**E**) after LPS incubation. Bars represent the mean of media cytokine detection (pg/ml) ± SD of three independent experiments. Counts were performed after 24 h of cell culture (**B**, **C**) or after 2, 4, or 8 h incubation with LPS (**D**, **E**). *p < 0.05, as compared to CTL cells (ANOVA followed by Bonferroni’s test).

Since nuclear MUC1-C is also known to interact with the pro-inflammatory transcription factor NF-κB (nuclear factor kappa B) p65 [40] and cytoplasmic YAP is needed to prevent TRAF6 (tumor necrosis factor receptor-associated factor 6)-mediated NF-κB activation [41], we checked endothelial activation dependent on PATJ levels, studying the expression of the adhesion molecules ICAM-1 (intercellular adhesion molecule-1) and CD44. ICAM-1 was highly upregulated in *PATJ* KD2 cells and downregulated in *PATJ* KD3, meanwhile CD44 was detectable in *PATJ* KD2 and highly expressed in *PATJ* KD3 cells (Fig. 6A). TRAF6 levels were also upregulated in *PATJ* KD3 cells. By contrary, the anti-inflammatory heat shock protein αβ−crystallin [42] was upregulated in *PATJ* KD1 and highly expressed in *PATJ* KD2 (Fig. 6A), suggesting that in these intermediate *PATJ* KD clones αβ−crystallin may contribute to maintain the transient EndMA phenotype. We next measured cytokines secretion in the culture media. Interestingly, only *PATJ* KD3 cells produced high levels of interleukin-6 (IL-6) under regular growth conditions (Fig. 6B), meanwhile IL-8 production was detectable in *PATJ* KD2 and KD3 cells (Fig. 6C). None of the other cytokines measured, IL-1β, IL-15, IL-18, and TNF-α, could be detected (data not shown). Once cells were challenged with lipopolysaccharide (LPS), all clones secreted equal amounts of both IL-6 and IL-8 after 6 h of incubation (Fig. 6D, E), demonstrating that all cells kept the ability to secrete both IL-6 and IL-8 once Toll-like receptor 4 is stimulated with LPS. Our results showed that only the *PATJ* KD3 cells with the mesenchymal phenotype constitutively secrete IL-6, probably as an autocrine or paracrine feedback loop to maintain the mesenchymal phenotype, as previously shown in other cellular systems [43–45].

### The EndMA transient state of intermediate *PATJ* KD clones favors cellular migration and tubular network formation

**Fig. 7:**
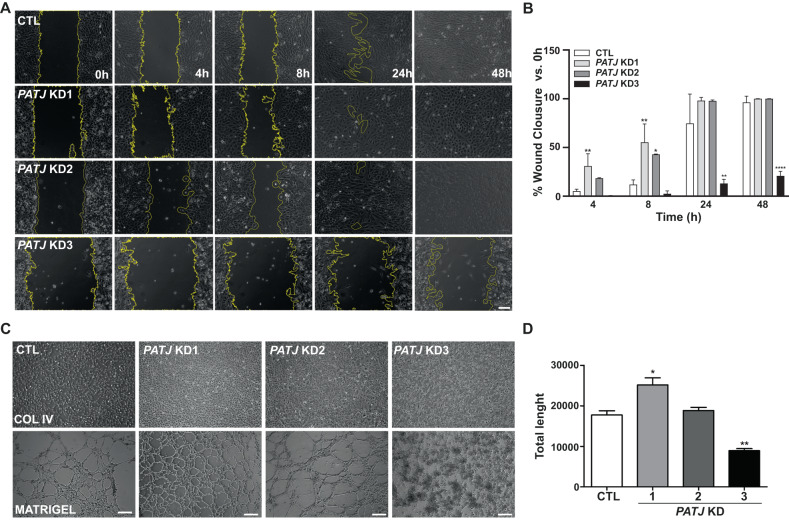
The EndMA state of intermediate *PATJ* KD cells favors cell migration and tubular network formation. **A**, **B** Wound healing assay showed that intermediate *PATJ* KD clones migrate significantly faster after 4 and 8 h post-scratch. **C**, **D** Intermediate *PATJ* KD clones retain the ability for tubulogenesis, meanwhile the *PATJ* KD3 with mesenchymal phenotype could not perform angiogenesis in vitro, as shown with the tubular network formation assay of cells grown on Matrigel (10 mg/ml). **D** Total tube length quantification of three independent experiments, measured with the customized computer analysis “Angiogenesis Analyzer” for the Image J Program. *p < 0.05; **p < 0.01 and ****p < 0.001 as compared to CTL cells (ANOVA followed by Bonferroni’s test).

Vascular regeneration is accompanied by vessel elongation, which is promoted by EC migration. During this process the expression of EndMT-related transcription modulators, such as ZEB1, are upregulated [37, 38]. Thus, the transient EndMA state is thought to exhibit reversible changes during vascular remodeling, such as transient occurrence and return to ECs [29]. To demonstrate that *PATJ* depletion exacerbates cellular migration, a scratch wound healing assay was performed. As expected, intermediate *PATJ* KD clones with the EndMA phenotype (KD1 and KD2) significantly increased cell migration 4 and 8 h after the scratch (Fig. 7A, B), meanwhile *PATJ* KD3 was unable to heal the wound during the 48 h that the experiment lasted, probably due to the severe depletion of key regulatory proteins of the cytoskeleton, as MLC and ARHGAP6 (Fig. 5J).

Cell tube formation assay was performed to prove that the EndMA state of intermediate *PATJ* KD clones allows cells to conserve the ability to organize forming tubular networks, once grown on Matrigel. In fact, *PATJ* KD1 had even improved sprouting ability than CTLs (Fig. 7C, D). By contrary, the mesenchymal *PATJ* KD3 cells, completely lost their potential for endothelial morphogenesis and were unable to form tubular networks. These results confirmed that the EndMA state of intermediate *PATJ* KD clones allows cells to migrate and tubulogenesis.

## Discussion

Genetic factors may determine different degrees of disability after an IS. Prior to this study we reported a genetic association between low frequency genetic variants in *PATJ* gene and worse IS functional outcome [2]. Here we explored the relationship between *PATJ* expression, stroke functional outcome and the underlying molecular mechanisms. Expression analysis in humans, rodents, and cultured ECs demonstrated that *PATJ* downregulates in response to ischemia/hypoxia and lower *PATJ* levels correlate with functional independence at 3 months in IS patients. To disclose the molecular benefits of silencing PATJ in ECs, we generated hCMEC/D3 clones *PATJ* KD.

hCMEC/D3 cells express 5 different isoforms of PATJ, three of high molecular weight (230, 200, and 135 kDa), generated by differential splicing [21], and two smaller species of 80 and 60 kDa. The 80 kDa species may be equivalent to the 75 kDa previously reported in CaCo-2 cells, suggested to have a different initiation of translation [21]. The characterized *PATJ* KD clones differed in their PATJ species patterns of expression. *PATJ* KD3, with severe depletion of all PATJ species, presented the classical hallmarks of EndMT, including the upregulation of mesenchymal markers, such as Vimentin, FSP1/S100A4 or tight junction proteins and the downregulation of epithelial markers as PECAM-1/CD31 and VE-cadherin [24, 46]. By contrary, intermediate *PATJ* KD clones (KD1 and KD2), which shared only a severe depletion of the 80 kDa-PATJ species, acquired a transient state of mesenchymal activation (EndMA) [25], with mixed endothelial and mesenchymal character, and a remarkable actin cytoskeleton rearrangement. EndMA has been implicated at the onset of angiogenesis [25, 47]. The transient state of EndMA allows cells to adopt tip cell peculiarities and revert it to endothelial properties. Tip cells that lead emerging sprouts lack apicobasal polarity, degrade both basal membrane and extracellular matrix and migrate into the extravascular milieu [25, 47]. The angiogenic transition is partial and reversed: the cells resume endothelial functions as nascent blood vessels take shape. Vascular sprout requires the integration of multiple signaling pathways, including TGFβ/BMP/SMAD signaling, Notch, PI3K/Akt, and Hippo. All of these signaling pathways, which are intimately related, were finely tuned in *PATJ* KD cells. Notch coordinates tip versus stalk cell fates within a growing sprout [48]. PI3K/Akt pathway, is also dynamically regulated during vessel sprouting. On the one hand, inhibition of PI3K/Akt signaling promotes sprouting of pre-existing vessels, by inducing EC elongation [49]. But on the other hand, after this first inhibitory phase, active PI3K/Akt supports vascular patterning by controlling EC rearrangement [50]. Moreover, induction of PI3K/Akt signaling by vascular endothelial growth factor A (VEGF) through its receptor leads to activation of YAP/TAZ [51]. Other upstream signaling cues such as blood flow shear stress and loss of apicobasal polarity also control YAP/TAZ angiogenic program [52]. After stroke, PATJ depletion triggers the loss of cell polarity that may explain YAP/TAZ translocation to the nucleus, which we observed it was accompanied by MUC1-C together with β-catenin and ZEB1. YAP/TAZ-β-catenin nuclear co-translocation has been described [53–55], associated to genes involved in EndMT and vascular patterning [54]. MUC1-C also binds to β-catenin promoting the formation of MUC1-C/YAP/β-catenin complexes in the nucleus [36]. MUC1 protects the apical border of polarized ECs as a component of the mucous barrier. Upon loss of polarity due to epithelial damage, MUC1 is proteolytically cleaved into an N-terminal extracellular subunit (MUC1-N) and a transmembrane C-terminal subunit (MUC1-C). This cleavage can be auto-proteolytically-mediated or induced by different proteases, including MT1-MMP [56], upregulated in *PATJ* KD cells. MUC1-C promotes the synthesis and secretion of VEGF through the PI3K/Akt pathway [57] and modulates the hypoxic response through regulating the expression, stabilization, and activity of HIF-1α [58]. MUC1-C can bind and activate different transcriptional modulators, including ZEB1 [38] and β-catenin [59] with whom induces EndMT. The pro-inflammatory phenotype observed in *PATJ* KD could also be attributed to MUC1-C, since it forms nuclear complexes with NF-κB p65 supporting expression of its target genes, including MUC1 itself [40, 60].

Based on our data, we propose a model in where after IS, PATJ is downregulated in ECs, and therefore Crumbs disassembles, YAP/TAZ is liberated, and cell polarity is lost. MUC1 is cleaved, MUC1-C accumulates in the cytoplasm, interacts with free YAP/TAZ and β-catenin, forming stable complexes that are recruited to the nucleus, where activate gene expression that accounts for EndMA.

Taken together, here we demonstrate that *PATJ* downregulates in response to IS. In ECs, *PATJ* depletion is beneficial because it translates into a sophisticated higher-order signaling reprogramming that promotes EndMA to activate angiogenesis. Fine modulation of EC polarity through *PATJ* downregulation could be a therapeutically strategy to stimulate angiogenesis after IS.

## Materials and methods

### Subjects selection

Acute ischemic stroke (IS) patients were selected from the prospective cohort of the GODS project [2]. Inclusion criteria were patients ≤65 years old with functionally independence before the stroke, with a stroke of anterior circulation territory, and an initial neurological severity with National Institute of Health Stroke Scale (NIHSS) ≥ 4. Functional outcome at 3 months was assessed with the modified Rankin Scale (mRS). *PATJ* expression analysis comparing mRS = 0–2 vs mRS = 4–5 was adjusted for NIHSS, stroke subtype, age, sex, smoking status, and principal components. All participants provided informed consent for data collection.

### RNA extraction from human leukocytes and *PATJ* mRNA quantification by real-time PCR (qPCR)

Different cohorts of patients have been used in this study to perform *PATJ* mRNA quantification. The RNA samples from the individuals of the GRECOS project [19] were extracted from isolated buffy coat using the RiboPureTM-Blood kits (Ambion, Woodward St. Austin, USA). qPCR on these samples was performed on a 7900 Real Time PCR System (Applied Biosystems, Foster City, CA, USA) using a TaqMan fluorogenic probe for PATJ (Hs00902482_m1) and normalizing with Cyclophilin A (PPIA, Hs99999904_m1) and Glyceraldehyde-3-Phosphate Dehydrogenase (GAPDH, Hs99999905_m1).

The RNA samples from the IS patients of the GODS cohort [2] were collected 24 h after IS using the PAXgene RNA tubes (Qiagen), and RNA extraction was performed using the PAXgene Blood RNA extraction Kit (Qiagen), following manufacturer’s protocol. qPCR was performed on a CFX96 Touch Real-Time PCR Detection System (Bio-Rad Laboratories, CA, USA) using SensiFAST SYBR No-ROX Kit (Meridian Biosciences, Memphis, TN, USA) according to the manufacturer’s instructions. *PATJ* primers were (Forward 5’-CACTCCACGAGTCATTCCTAAC-3’ and Reverse 5’-GAGCAGTTCCTTGCCTCTTT-3’). *GAPDH* endogenous control primers were (Forward 5’-ATGTTCGTCATGGGTGTGAA-3’ and reverse 5’-ACCACCAACTGCTTAGCACC-3’). Relative mRNA expression levels for the genes of interest were measured using the Pfaffl method [61].

### Focal transient cerebral ischemia

C57BL/6 mice (7 males between 7 and 9 weeks) were purchased from Janvier laboratories (Saint Berthevin, France). Transient occlusion of the distal part of the MCA was performed as previously described [62]. Briefly, mice were anaesthetized with isofluorane via facemask (4% for induction, 2% for maintenance in air, and 79% N2/21% O2; Abbot Laboratories, Madrid, Spain), temporal muscle was retracted and a small craniotomy in the left temporal bone was performed to expose the MCA. Finally, the distal portion of the artery was compressed with a 30-G needle for 60 min and allowed to reperfuse for 48 h when mice were euthanized under deep anesthesia, transcardially perfused with cold saline and brains removed to obtain 1 mm slices, which were stained with TTC [62] to verify cortical infarct lesions and later snap frozen in dry ice as ipsilateral (IL) and contralateral (CL) brain hemispheres for further analysis. Cortical cerebral blood flow was monitored during occlusion and reperfusion.

Later, IP and CL brain samples were used for RNA isolation with the PARIS TM kit (Invitrogen, CA, USA), following the manufacturer’s procedure. The quality and quantity of RNA were measured using Nanodrop Spectrophotometer.

### Cells, culture conditions, OGD, and chemical hypoxia

HEK293T cells were grown in DMEM medium supplemented with 4 mM glutamine, 10% FBS, and 100 units/ml penicillin/streptomycin.

Immortalized human cerebral microvascular endothelial cells (hCMEC/D3) originally isolated from microvessel fragments of the temporal lobe of an adult with epilepsy [5] were grown in flasks coated with rat tail collagen type I high concentration (Corning, NY, USA) with endothelial cell basal medium-2 (EBM-2; Lonza, Walkersville, MD, USA) supplemented with 2% fetal bovine serum (FBS), hydrocortisone, human basic fibroblast growth factor, vascular endothelial growth factor, R3-insulin-like growth factor-1, ascorbic acid, human epidermal growth factor, Gentamicin sulfate-Amphotericin (GA-1000) and heparin (EGM-2 SingleQuots Supplements; Lonza, Walkersville, MD, USA).

Oxygen-glucose deprivation (OGD) was performed as done before [63]. OGD medium consisted in RPMI 1640 without glucose (Lonza), and without FBS and growing factors. In addition, OGD medium was deprived of oxygen for 24 h prior to use. The normoxia medium was RPMI 1640 (Sigma), supplemented with 2 g/L glucose and free of serum and growing factors. hCMEC/D3 cells were subjected to OGD treatment for 5- and 10 h with OGD medium in a hypoxic chamber (INVIVO2 200, Ruskinn UK) with 0.5% O_2_, 5% CO_2_, N_2_ and 100% humidity.

Chemical induced hypoxia was achieved by incubating hCMEC/D3 cells for 8, 24, or 48 h in EBM-2 medium supplement with the HIF-1α stabilizing compound (Sigma) cobalt chloride (CoCl_2_: 150 µM).

### Generation of *PATJ* knockdown hCMEC/D3 cells by lentiviral infection with *PATJ* shRNA

Lentiviral particles carrying *PATJ* shRNA were generated with the Trans-Lentiviral shRNA Packaging Kit using Calcium Phosphate Transfection Reagent, following the manufacturer’s instructions (Dharmacon, Lafayette, CO, USA). The mature antisense target sequences used were TATGGTTCATCTCTAAATC and TGAATGGTATGATGAAGCT. Virus titer was determined by reporter (TurboGFP fluorescent) expression, obtaining values between 1.57 × 10^5^ and 1.03 × 10^6^ transducing units (TU)/ml.

For lentiviral particles infection, hCMEC/D3 cells (5 × 10^5^ cells/well) were seeded into 10-cm Petri dishes 1 day before infection. 1 viral particle/cell was used to infect the target cells for 72 h. Cell lines were subjected to FACS sorting to select by fluorescence, and single-cell were seeded per well in a 96-well plate. Control (CTL) cells were subjected to infection with lentiviral particles harboring scramble shRNA, following the same procedure.

### Endothelial transwell permeability assay

To perform permeability assays, hCMEC/D3 cells were seeded on collagen and fibronectin-coated transwell inserts (12-well, transwell polyester membrane inserts, pore size 0,4 μm; Corning, NY, USA) at 2 × 10^5^ cells/ml. After three days in culture, being reached confluence, the insert culture media was replaced by 500 μl of phenol red-free DMEM containing 2 mg/mL of 70 kDa Fluorescent isothiocyanate (FITC)-dextran. Then, inserts were changed to 12-well plates containing pre-warmed DMEM and sequentially transported to new wells at 5 min intervals for 30 min in total. During the process, cells were maintained at 37 °C. The fluorescence of each well was then measured at 485/520 nm (ex/em), and the apparent permeability coefficients were calculated from curve slopes fitted to linear regression. Permeability (Pe) coefficients in *PATJ* KD cells were expressed as % Pe compared to CTL cells.

### MTT assay

The MTT (3-(4,5-dimethylthyazol-2-yl)2,5-diphenyl-tetrazolium bromide) reduction assay was performed to assess possible viability differences between CTL and *PATJ* KD cells. 24 h prior the assay, 5 × 10^4^ hCMEC/D3 cells were plated in 24-well culture plates and incubated under normoxia conditions (20% oxygen). MTT solution was added to a final concentration of 0.5 mg/ml to each well and incubated for 1 h at 37 °C. Then, the medium was replaced by DMSO to dissolve the formazan blue precipitate formed and quantified at 560 and 620 nm in a microplate reader (Synergy HT with data analysis software KC4; Bio-Tek Instruments Inc, Winooski, VT, USA).

### RNA cell extraction and reverse transcription-quantitative PCR (RT-qPCR)

Total RNA was extracted from scrambled CTL and *PATJ* KD hCMEC/D3 cells using QIAzol Lysis Reagent from miRNeasy Mini Kit (Qiagen, Hilden, Germany). RNA concentration and purity were measured using a Nanovue Plus (Biochrome Ltd, USA). cDNA was synthesized with SensiFAST™ cDNA Synthesis Kit (Meridian Biosciences), and qPCR was performed on a CFX96 Touch Real-Time PCR Detection System (Bio-Rad Laboratories, CA, USA) using SensiFAST™ SYBR No-ROX Kit (Meridian Biosciences), proceeding as explained above for human leukocytes.

### Gene expression array

Prior to analysis, RNA integrity and purity were determined using the RNA Nano 6000 Chip on an Agilent Bioanalyzer (Agilent Technologies Inc., Santa Clara, CA). All RNA samples showed high quality since RNA Integrity Numbers detected by Bioanalyzer were between 9 and 10. Probing of Affymetrix GeneChip Human Clariom S microarrays (Affymetrix UK Ltd., High Wycombe, UK) was performed as described in the manufacturer’s protocols. The probes were prepared from 100 ng aliquots of total RNA in accordance with the instructions supplied with the Affymetrix WT PLUS Reagent Kit. After 16 h hybridization, the arrays were washed and stained using the Affymetrix GeneChip Fluidics Station 450 and scanned at 0.7 μm resolution using the Affymetrix GeneChip Scanner 3000 7G. Analysis of gene expression data including normalization by the SST-RMA algorithm, probe summarization and control of data quality was performed using Transcriptome Analysis Console (TAC) Software 4.0.2 (ThermoFisher). Differentially regulated genes were identified based on an absolute fold change cut-off > 2.0 and an adjusted *P*-value < 0.05. Gene ontology pathways analyses were performed using ToppGene [64], Reactome [65], Panther [65], and WikiPathways [66].

### Cellular fractionation, western blotting (WB) and antibodies

Cytosolic and nuclear fractions were separated from 7 × 10^6^ hCMEC/D3 cells by whole-cell homogenization in Mitobuffer (0.25 M Sucrose; 0.2 mM EDTA and 10 mM TRIS) on ice using a 2 ml KIMBLE dounce grinder (Sigma-Aldrich). Lysates were centrifuged at 1000 × *g* for 15 min at 4 °C. This process was repeated three times. Collected supernatants (cytosol) were centrifuged at 10,000 × *g* for 15 min at 4 °C to eliminate cell debris. Pellets (nuclear fraction) were washed three times in Mitobuffer.

For WB analysis, fifty micrograms of hCMEC/D3 cell lysates proteins were resolved by NuPAGE 4–12% Bis-Tris Gel (Invitrogen) electrophoresis and immobilized on polyvinylidene fluoride (PVDF) membranes 0.45 µm pore size (Merk, Darmstadt, Germany). Primary antibodies: rabbit (rb) anti-Patj [21] was provided by A. Le Bivic (Aix-Marseille University, CNRS, Developmental Biology Institute of Marseille (IBDM), Marseille, France); rb anti-Akt, mouse (ms) anti-GSK3β, rb anti-VE-Cadherin, rb anti-ZEB1, rb anti-YAP and anti-YAP/TAZ, rb anti-ICAM-1, rb anti-CD44, rb anti-TRAF6, rb anti-αβ-Crystallin, rb anti-Histone 3 (H3), and rb anti-GAPDH, purchased from Cell Signaling as well as the Matrix Remodeling Antibody Sampler Kit to detect the MMPs and TIMPs; rb anti-β-catenin (D10A8), rb anti-Vimentin and rb anti MUC1-C, purchased from ABclonal; rb anti-ZO-1, ms anti-Occludin, rb anti-Claudin11 and rb anti-ARHGAP6 purchased from Invitrogen; rb anti-CD31 purchased from Proteintech; ms anti-Cytocrome C purchased from Novus. Secondary antibodies: anti-rb 800CW and anti-ms 700CW (LI-COR Biosciences). Proteins were visualized with fluorescence using Odyssey Imaging Systems (LI-COR Bioscience, Lincoln, NB, USA). The quantification of the bands was obtained with ImageJ software (National Institutes of Health, NIH).

To inhibit the proteosome, hCMEC/D3 cells were incubated with the inhibitor MG-132 (Sigma-Aldrich, San Louis, Missouri) for 3 h at 10 µM, prior to cell collection.

### Immunofluorescent labeling and confocal microscopy

5 × 10^4^ hCMEC/D3 cultured cells were fixed for 15 min in 4% paraformaldehyde. 1% BSA in PBS was used to block non-specific binding. Immunoassays were performed for 1 h at 30 °C with diluted primary antibodies, rb polyclonal ZO-1 (1:100, Sigma-Aldrich), rb monoclonal YAP (1:250, Cell Signaling), and ms monoclonal β-tubulin (1:300, Proteintech). Nuclei were stained with DAPI and samples were analyzed using a Zeiss LSM710 Laser Scanning Confocal Microscope (Carl Zeiss Microimaging, Inc.). Z-sections were acquired (0.7-μm interval), and final images were obtained after merging planes. Images were processed using Zeiss LSM Blue software.

### Measurement of the G-actin/F-actin ratio

F-actin and G-actin were evaluated in hCMEC/D3 cells using a G-actin/F-actin in vivo assay kit (BK037; Cytoskeleton Inc., Denver, CO, USA) following the manufacturer’s instructions. Actin quantification was performed via SDS-PAGE and WB analysis using the anti-G-actin antibody provided with the kit.

### Cytokines quantification

4 × 10^5^ *PATJ* KD or CTL hCMEC/D3 cells were seeded in a 24-well plate. Culture media was collected after 24 h (basal, t = 0) and after 2, 4, and 6 h incubation with 100 ng/ml LPS. Cytokines levels were measured by Luminex Multiplex Assay (HSTCMAG-28SK; Merck KGaA, Darmstadt, Germany) using 6 plex Luminex Human Magnetic Assay kit, according to the manufacturer’s protocol. The tested 6 plex panel consists of the following chemokines: IL-1ß, IL-6, IL-8, IL-15, IL-18, and TNF-α.

### Monolayer wound healing assay

Cells were grown to full confluence in 24-well plates in complete EGM-2 media (Lonza) and were incubated overnight. Cell cultures were scratched with a 10 µL sterile pipette tip and extensively washed with PBS to remove detached cells and debris. One scratch per well was generated, and then center-imaged at 5× magnification, using a Zeiss Axio Observer 200 M microscope equipped with a Zeiss AxioCam MRm camera with maximum contrast (Carl Zeiss AG, Feldbach, Switzerland). Cells were then incubated in serum-reduced or full-growth medium. Images were taken at 0, 2, 4, 8, 24, and 48 h after the scratch. The wound areas were calculated and analyzed with Image J, version 1.8.0 (NIH, Bethesda, MD, USA) and expressed as a percentage of the day 0 value.

### Endothelial cell tube formation assay

The neovascularization assays were performed in 24-well plates coated with Matrigel (Corning, NY). hCMEC/D3 cells (3 × 10^5^) were plated on Matrigel and incubated for 18 h. Tube formation was observed at 5X using an inverted fluorescence Cell Observer microscope (Carl Zeiss, Jena, Germany). Length of tubes was measured in four independent fields using the “Angiogenesis Analyzer” software from Image J (National Institutes of Health).

### Statistical analysis

The SPSS 20.0 package (IBM Corporation, Armonk, NY, USA) and GraphPad Prism 6 (GraphPad Software, La Jolla, CA, USA) were used for statistical analyses. The normality was assessed by the Shapiro–Wilk test. In normally distributed variables, the significant differences between groups were assessed using Paired or Unpaired Student t-test and one-way analysis of variance (ANOVA) with Bonferroni’s post hoc test when appropriate. Statistical significances were accepted at *p* < 0.05 (*p < 0.005, **p < 0.001, ***p < 0.0001). Ene 3.0 software (www.e-biometria.com/) was used to calculate statistical power of our results and sample size (setting the power at 80% and level of significance at 0.05).

### Supplementary information

Original Data File

Supplementary Material

## Data Availability

All genomics data produced for this study have been deposited at GEO under accession number GSE237919. All other data needed to evaluate the conclusions in this paper are available in the Article and its Supplementary Information. Source data are provided with this paper.
