# Supplementary material for: Role of PATJ in stroke prognosis by modulating endothelial to mesenchymal transition through the Hippo/Notch/PI3K axis
Source: Cell Death Discov. 2024 Feb 17;10:85. doi: 10.1038/s41420-024-01857-z (PMC10874379; doi:10.1038/s41420-024-01857-z)
Supplement: Supplementary file 1 — Original Data File [file 41420_2024_1857_MOESM1_ESM.docx]

**Fig. 1E**

1^st^ Blotting:

**
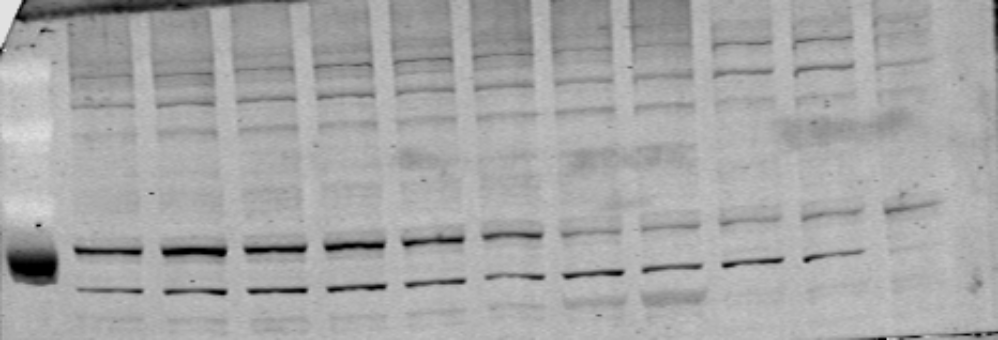
 kDa**

**PATJ**

250

150

100

75

**
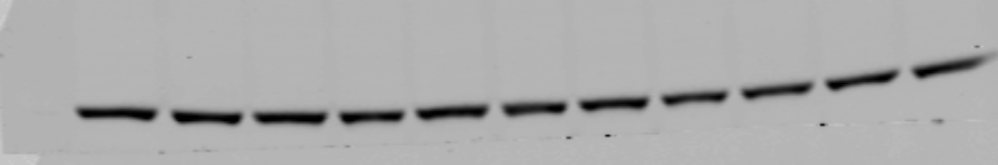

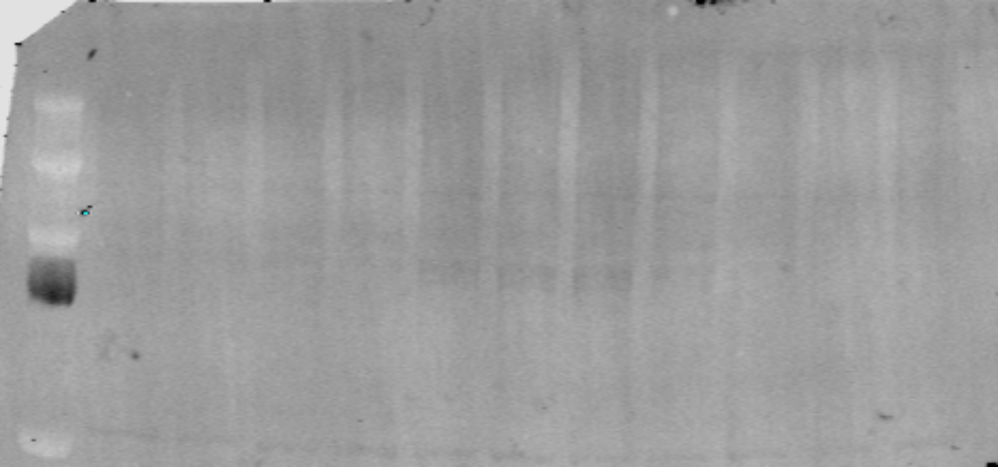
**

**GAPDH**

3^rd^ Blotting:

75

100

150

250

**HIF1-α**

2^ond^ Blotting after stripping

**
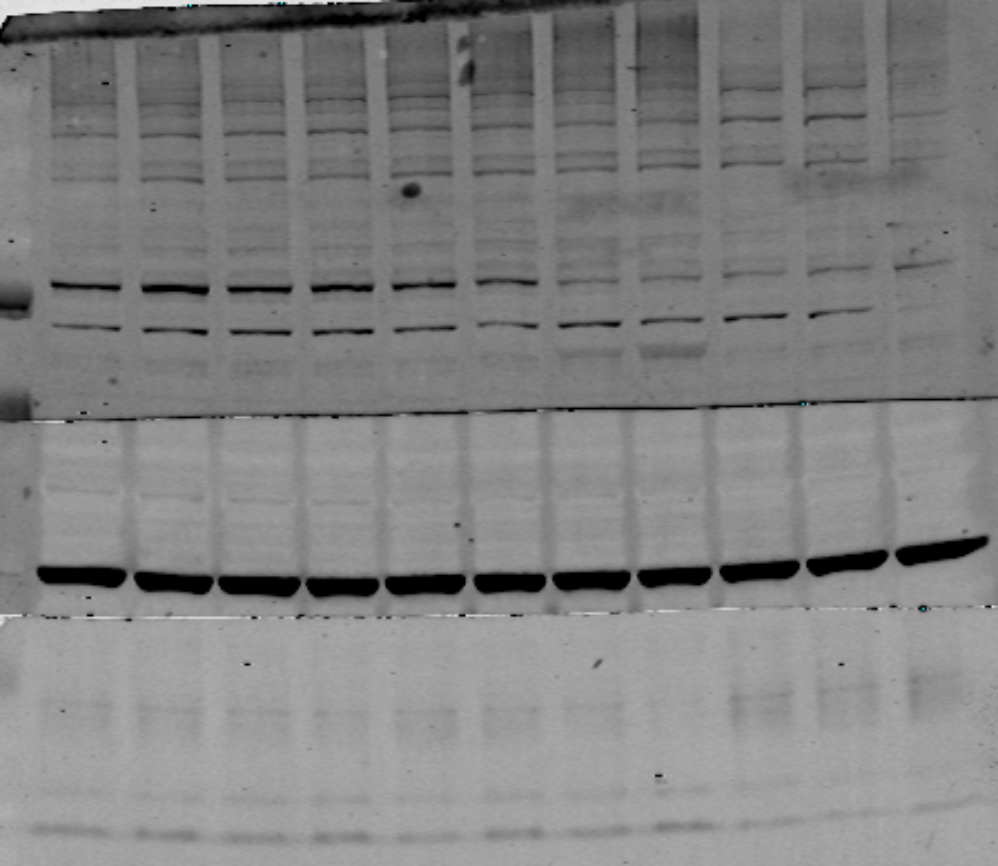
 kDa**

250

**PATJ**

150

100

**Fig. 2A**

25

**MUC1-C**

**αβ-crystallin**

**GAPDH**

37

50

75

**
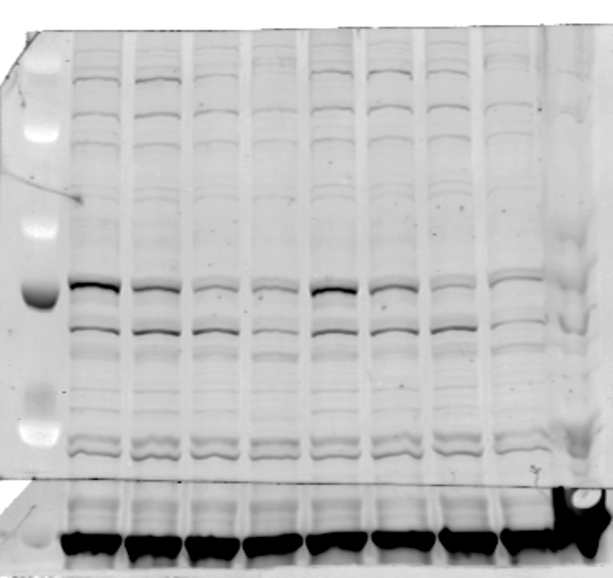
**

**kDa**

250

150

**PATJ**

100

75

50

**GAPDH**

37

**Fig. 2 D**


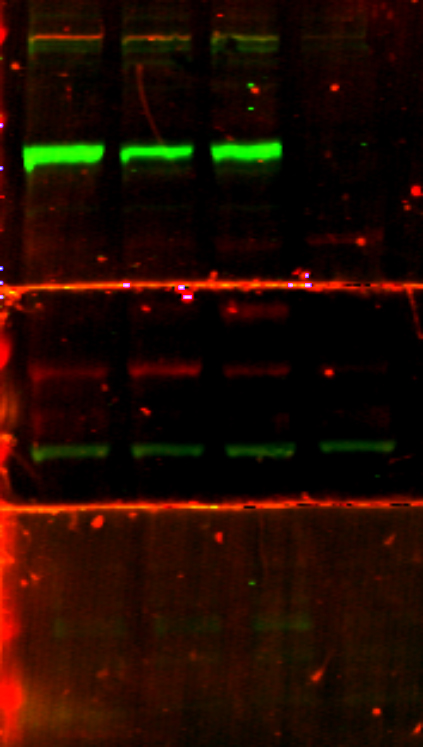


15

20

37

50

**kDa**

**Claudin 11 (Rb)**

150

250

**GAPDH (Rb)**

**OCCLUDIN (Ms)**

**ZO-1 (Rb)**

**CLAUDIN-11 (Rb)**

20

37

50

Rb= Green

Ms= Red

**Fig. 4A**

**
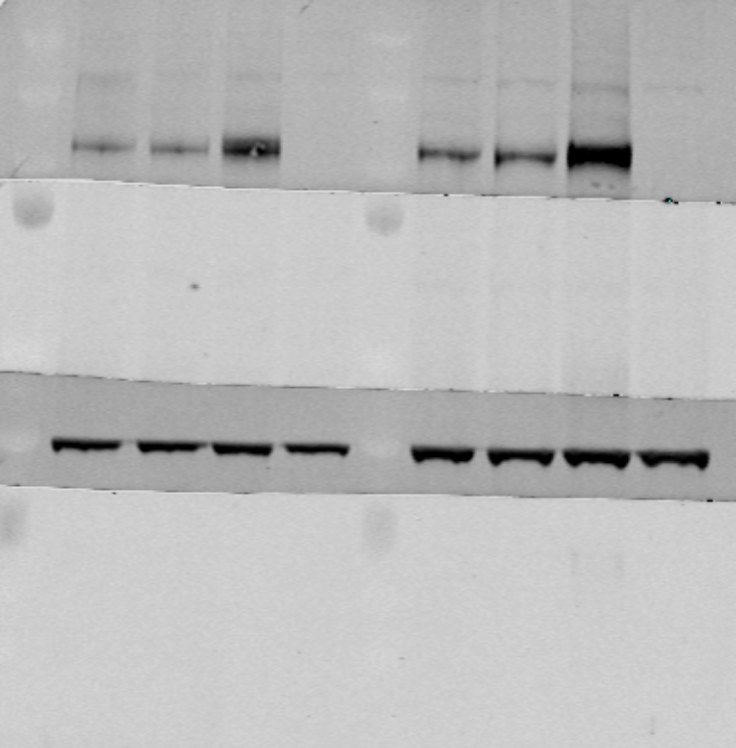
**

**kDa**

1^st^ Blotting:

**PECAM1**

**VE-CADH**

**VIMENTIN**

**GAPDH**

250

150

100

75

37

50

**
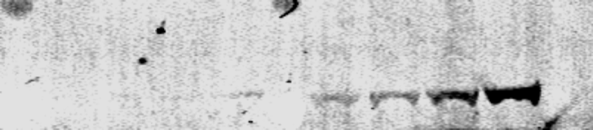
**

75

2^ond^ Blotting:

**VIMENTIN**

**Fig. 4B**

**
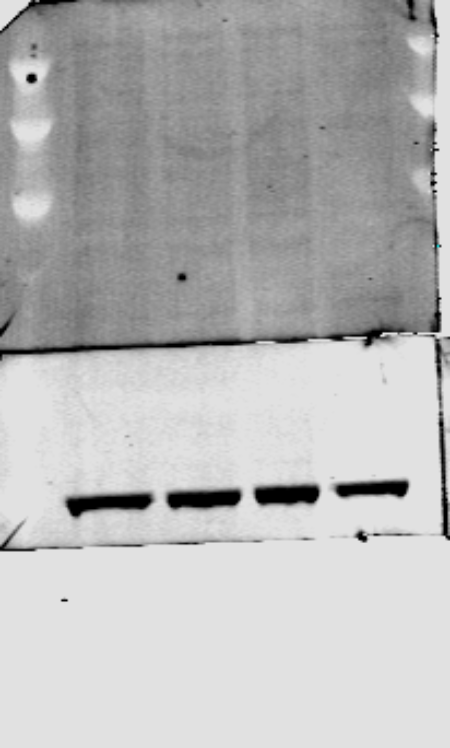

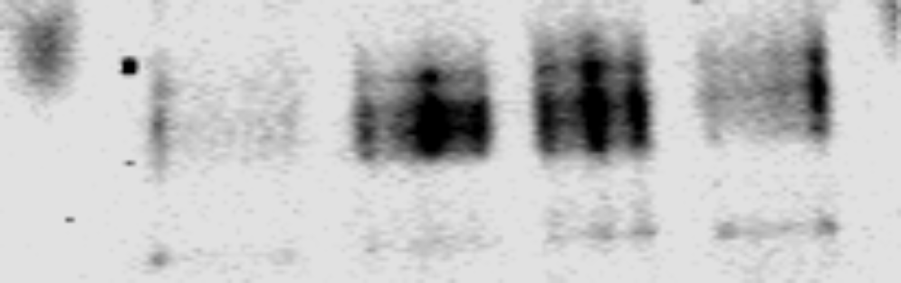
**

2^ond^ Blotting:

1^st^ Blotting

37

75

100

150

250

25

**kDa**

**GAPDH**

**MUC1**

**Fig. 4C**

**kDa**





**MMP2**

**MMP9**

**MT1-MMP**

**MMP3**

**TIMP3**

**TIMP1**

**TIMP2**

**MMP7**

100

75

60

50

37

25

20

15

**Fig. 4D**

**
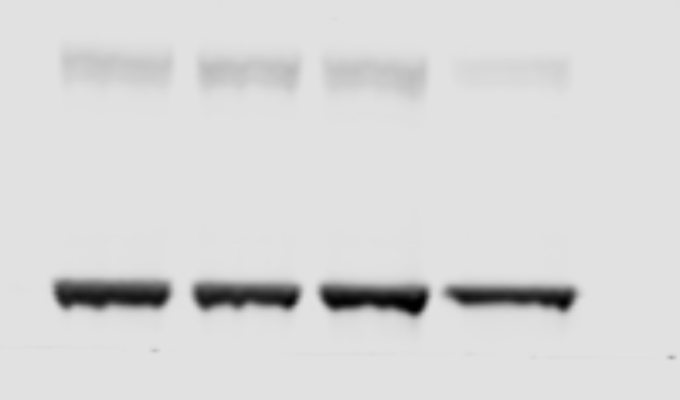

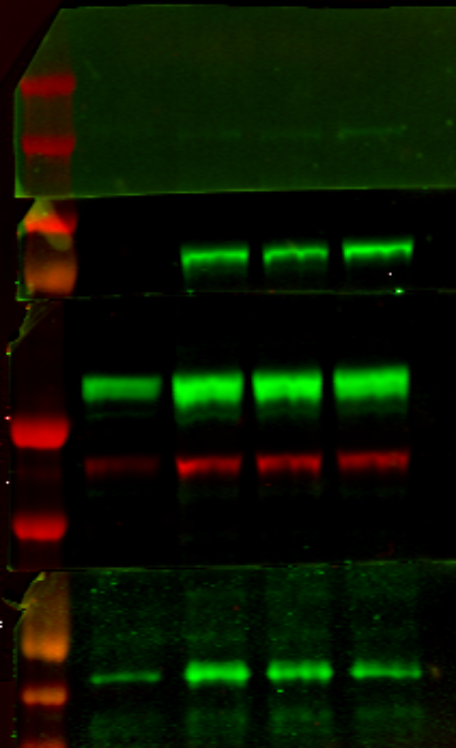
**

**GAPDH**

37

2^ond^ Blotting:

1^st^ Blotting:

**kDa**

**MLC (Rb)**

20

25

**ZEB1 (Rb)**

**GSK3B (Ms)**

**AKT (Rb)**

**B-CATENIN (Rb)**

37

50

75

100

150

250

**Fig. 4E**

**
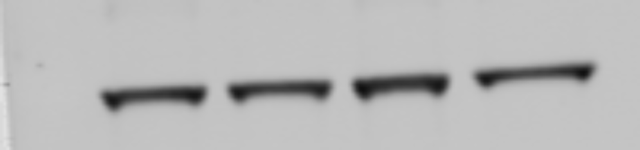

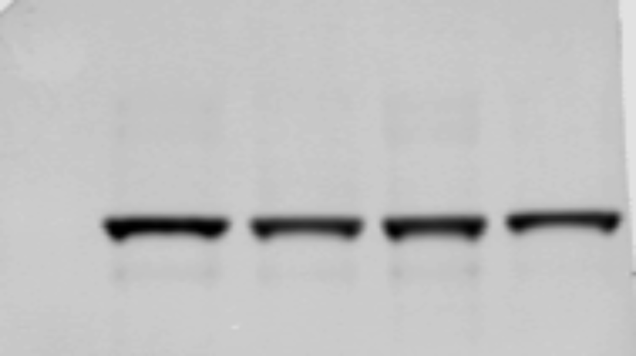

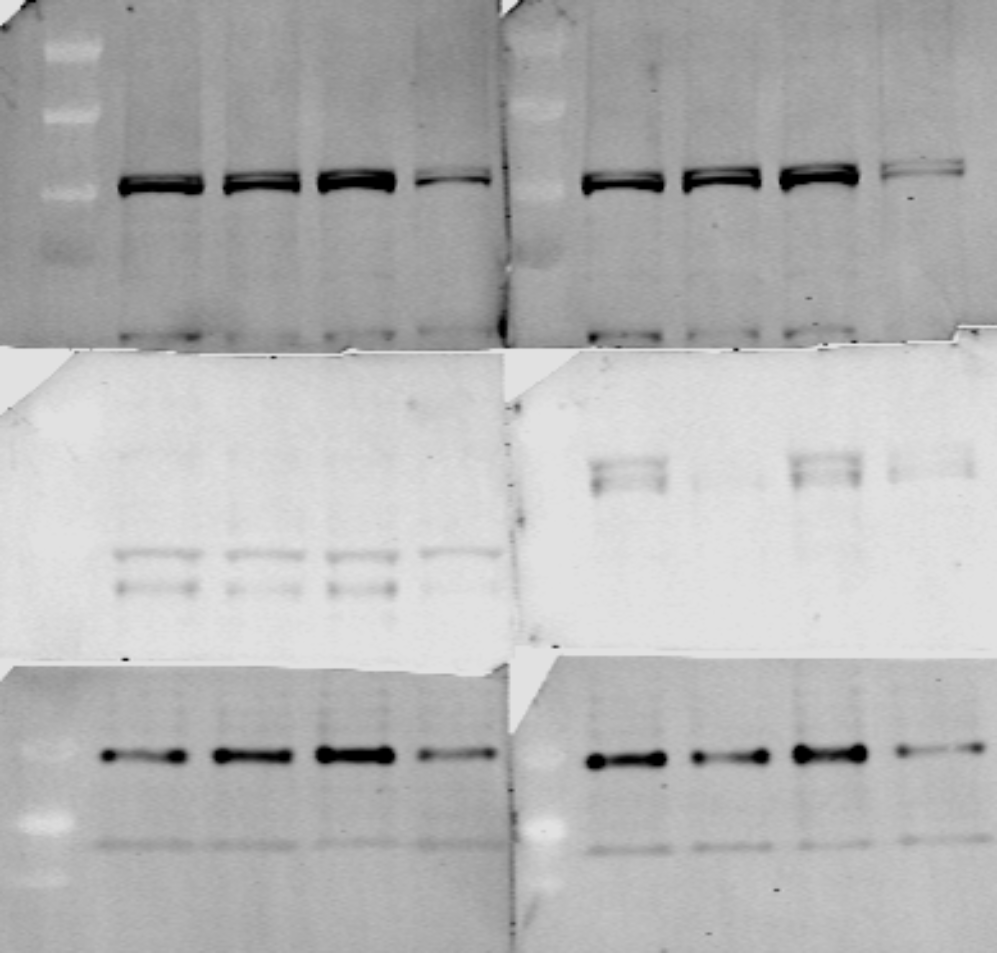
**

**kDa**

75

50

2^ond^ Blotting:

**MLC**

**RUNX3**

**HES1**

**ARHGAP6**

1^st^ Blotting:

15

20

250

150

100

37

37

**GAPDH**

**Fig. 4F**


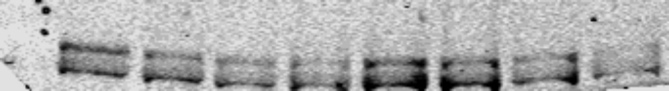

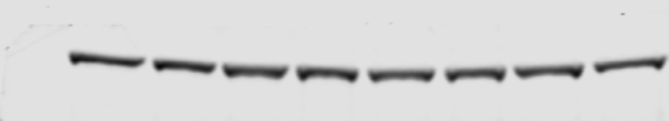
**
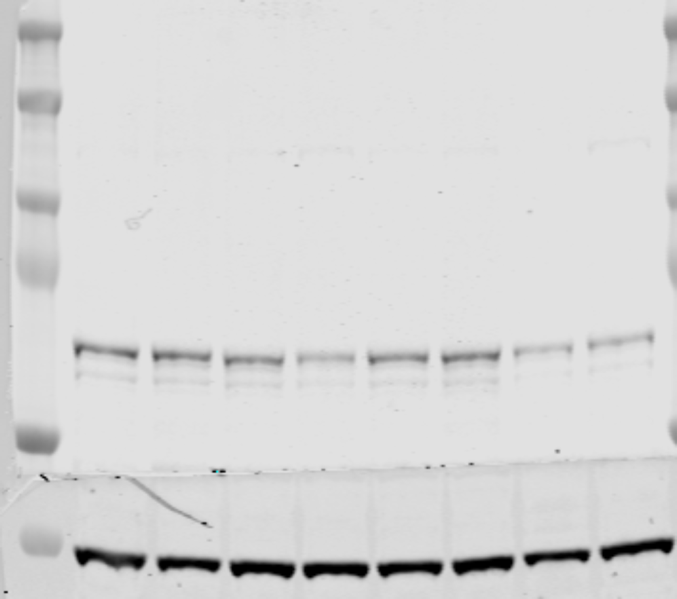
**

1^st^ Blotting:

2^ond^ Blotting:

37

50

37

50

**kDa**

75

100

150

250

**TAZ**

**GAPDH**

**GAPDH**

**YAP**

**Fig. 5A (Upper Pannel)**

**
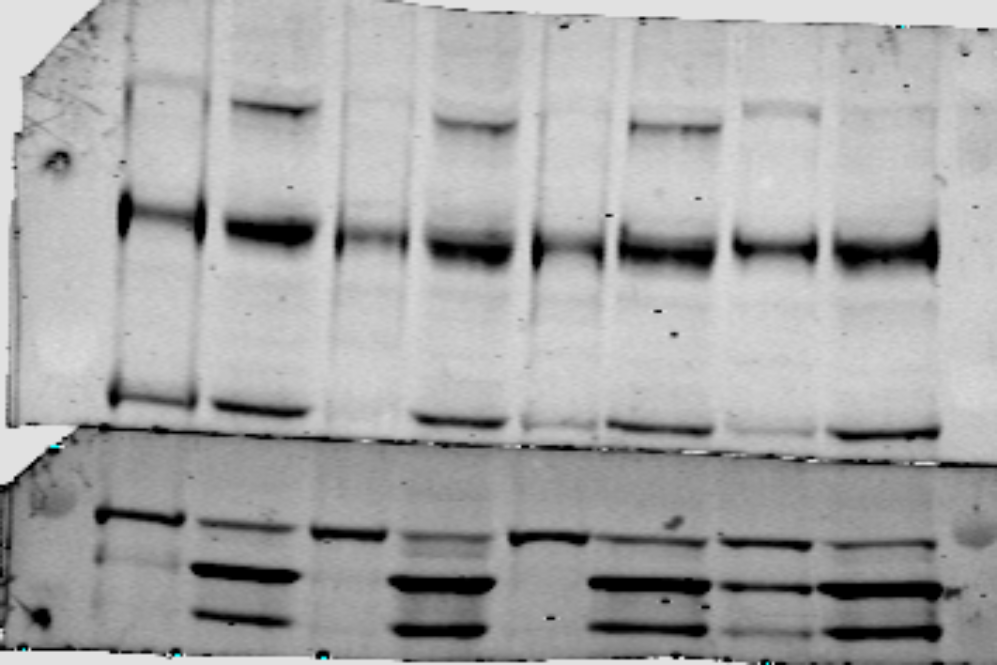
**
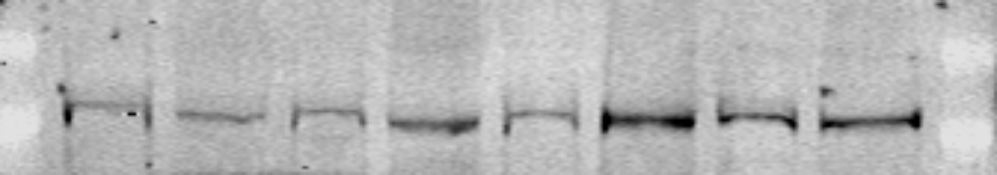


**VDAC**

**H3**

**TAZ**

**YAP**

**B-CATENIN**

25

**kDa**

250

50

37

75

100

150

**ZEB1**

**Fig. 5A (Lower Pannel)**
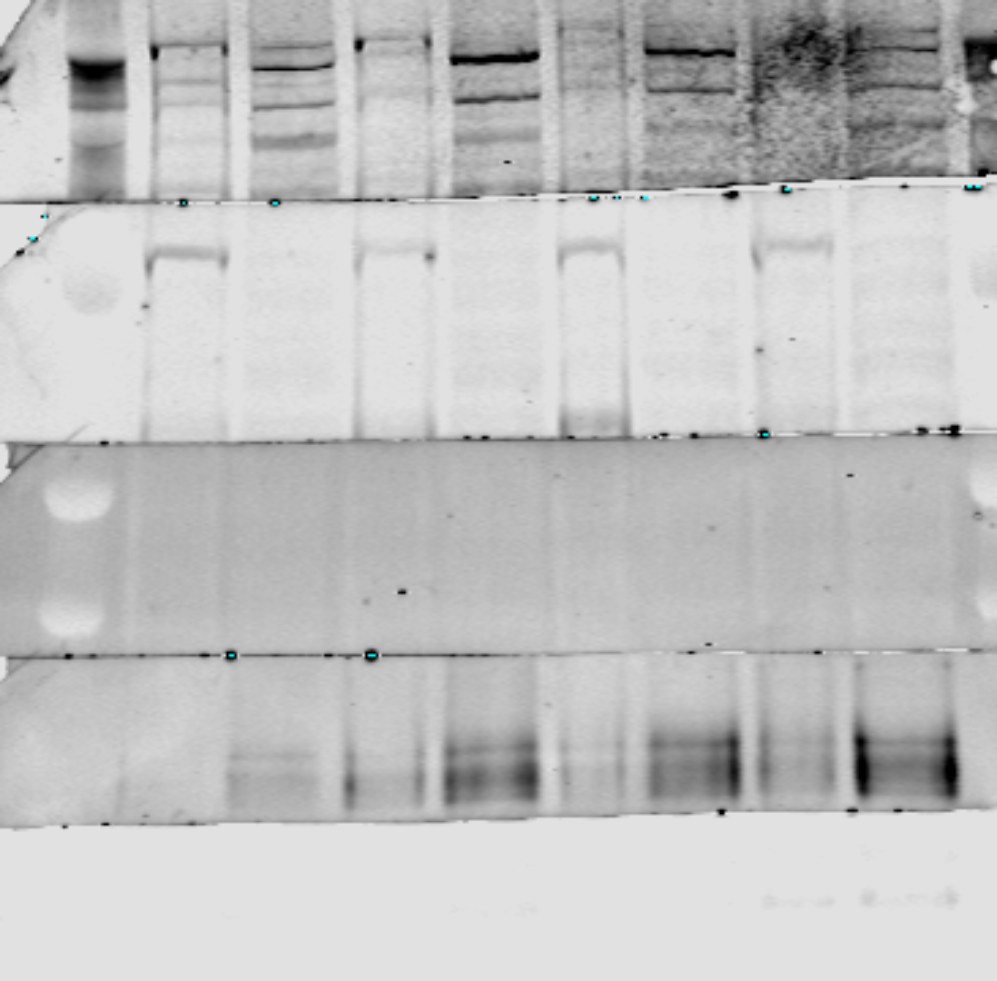

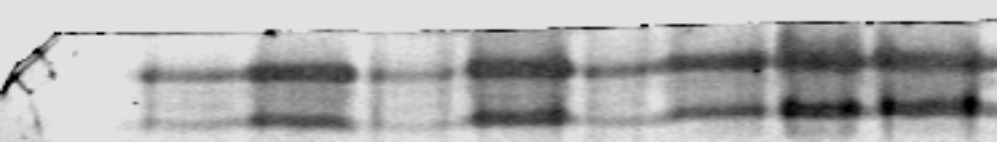

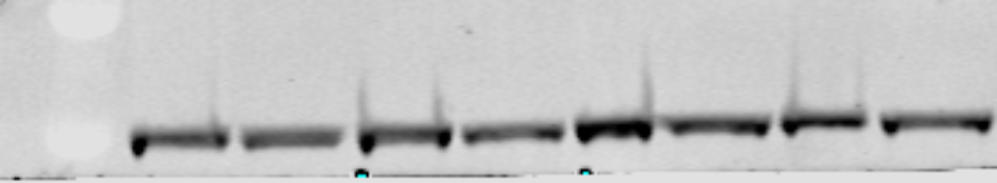


150

75

50

37

50

37

20

1^st^ Blotting:

2^ond^ Blotting:

**LIMK**

**MUC1**

**GAPDH**

**H3**

25

**kDa**

**Fig. 5H**

**
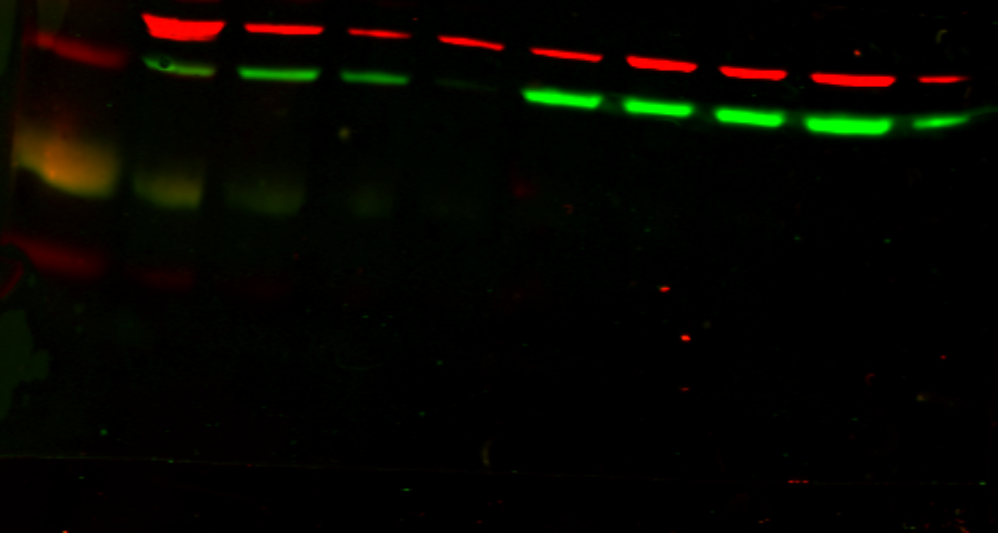

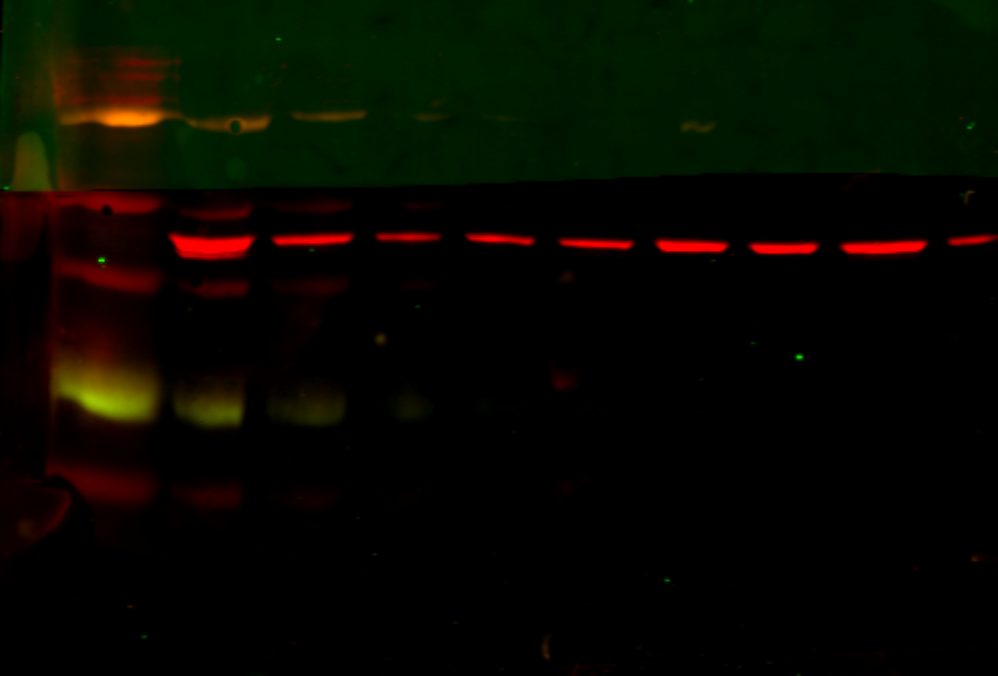
**

75

**kDa**

37

50

37

100

**GAPDH (Rb)**

**ACTIN (Ms)**

**PATJ (Rb)**

Rb= Green

Ms= Red

**Fig.5J**


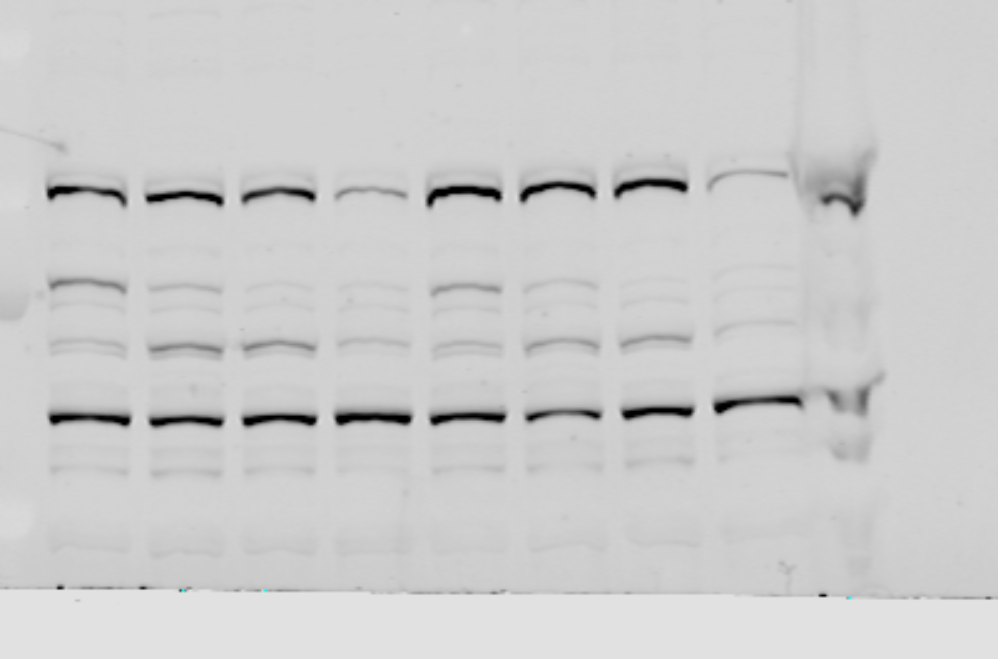
**
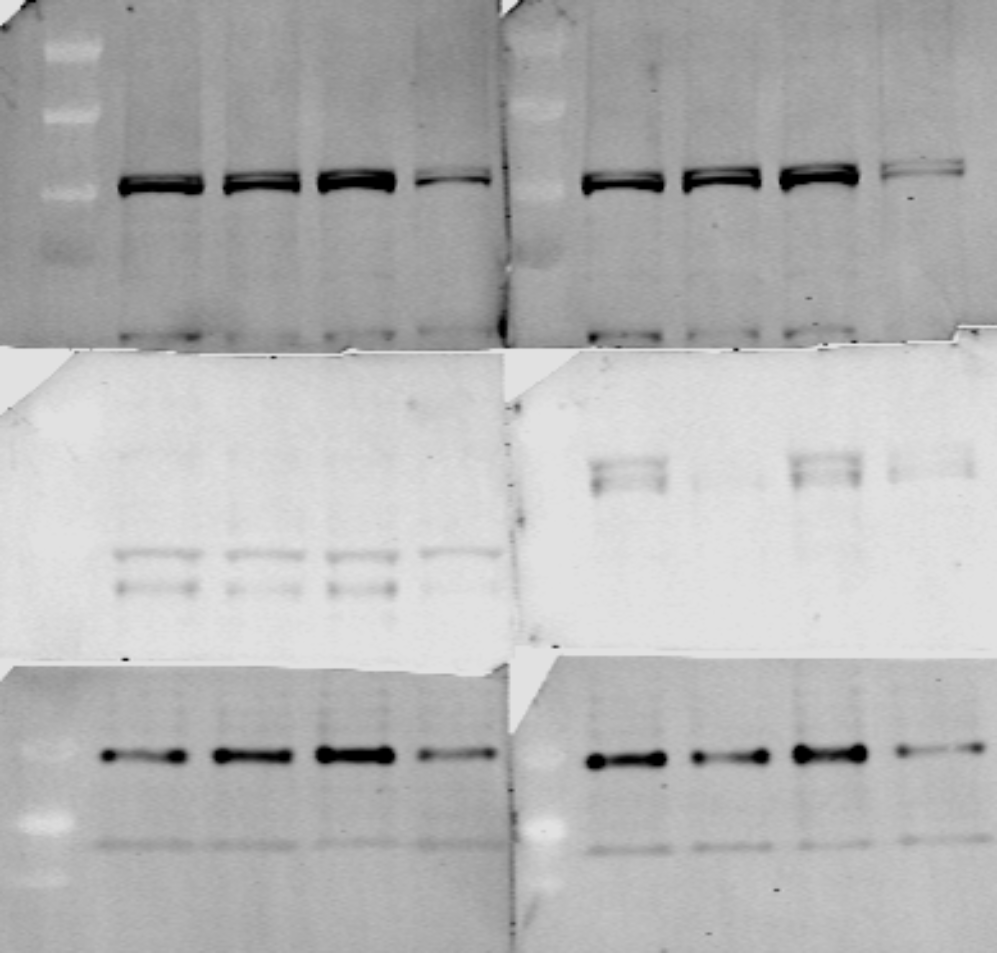
**

**ARHGAP6**

250

150

100

60

100

**kDa**

75

10

15

20

**HSP60**

**PATJ**

**ARHGAP6**

**MLC**

**Fig.6A**

**
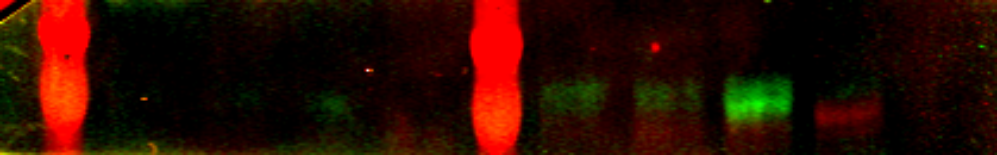

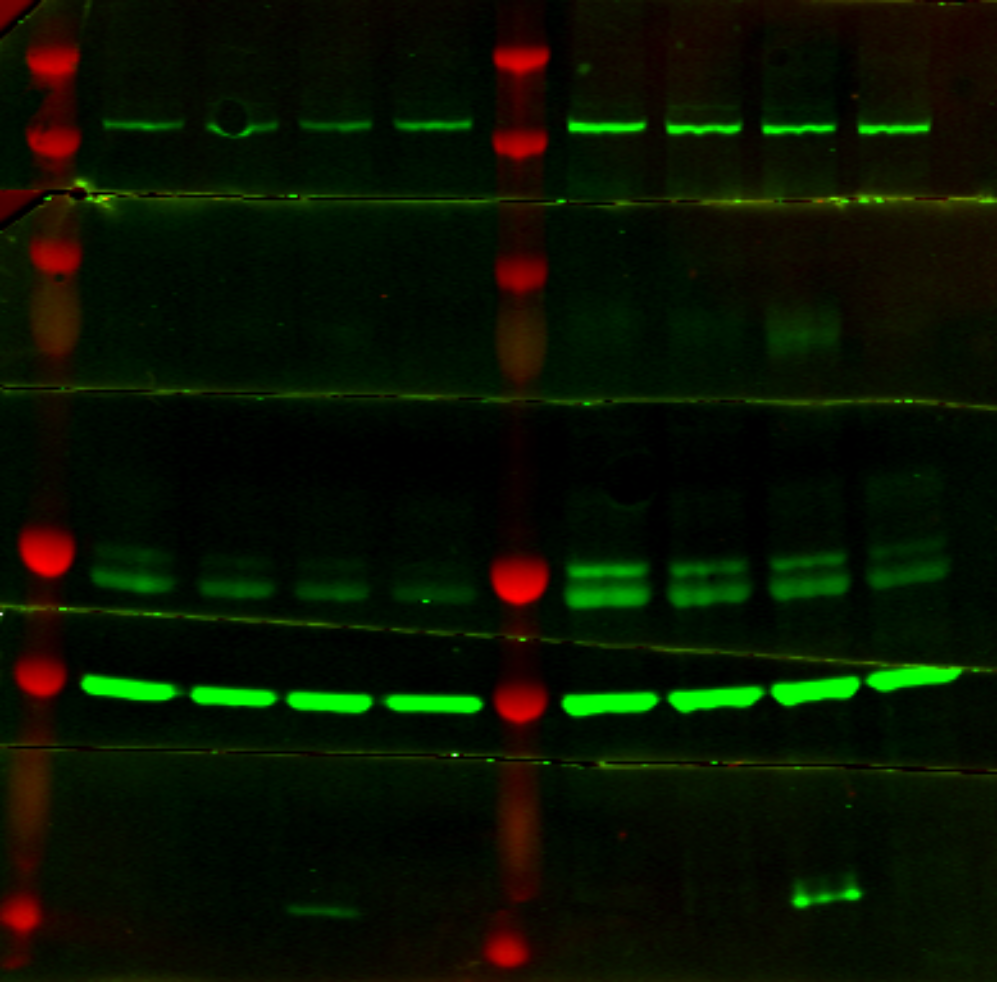
**

**TRAF6 (Rb)**

75

250

150

**kDa**

100

37

20

100

50

75

**CRYAB (Rb)**

**ICAM1 (Rb)**

**ICAM1 (Rb)**

**GAPDH (Rb)**

**CD44 (Ms)**

Rb= Green

Ms= Red

**Fig.6A**


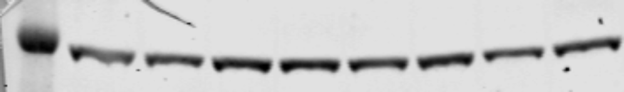

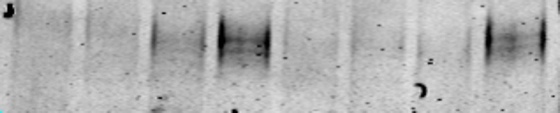
**
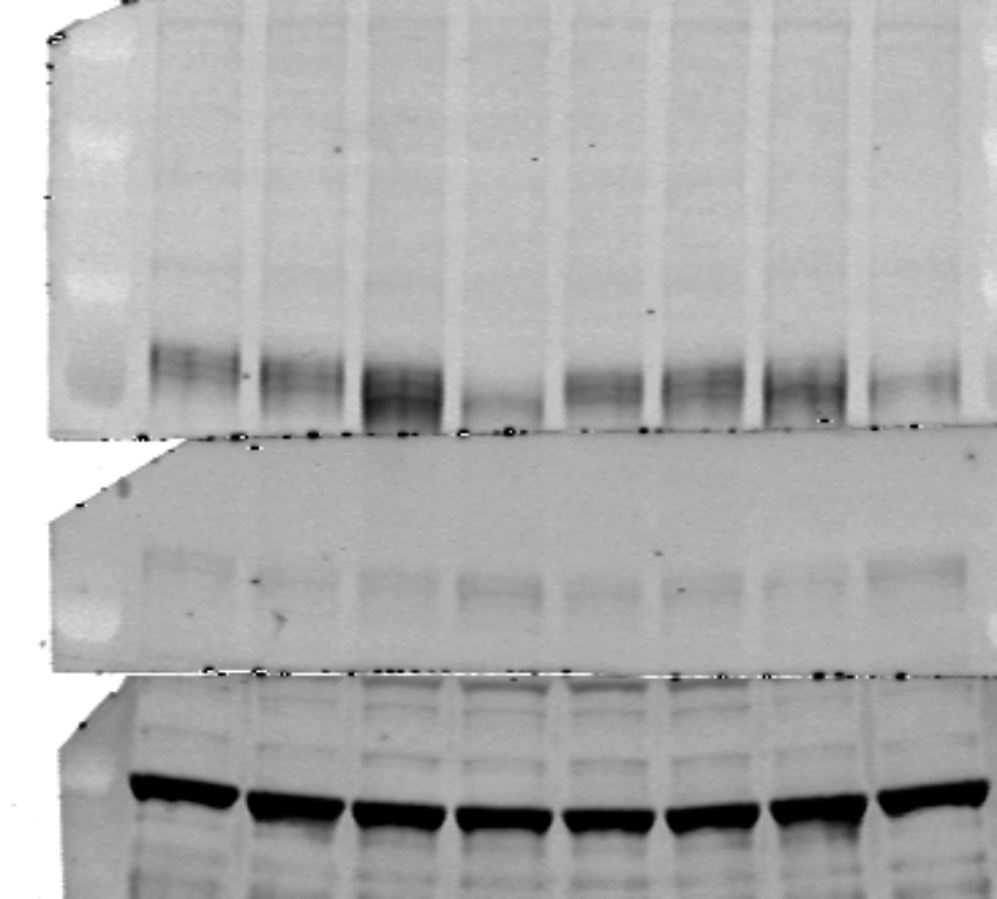
**

150

50

37

100

75

**kDa**

75

**GAPDH**

**CD44**

**ICAM1**

**TRAF6**

**GAPDH**

37
