# Supplementary material for: Role of PATJ in stroke prognosis by modulating endothelial to mesenchymal transition through the Hippo/Notch/PI3K axis
Source: Cell Death Discov. 2024 Feb 17;10:85. doi: 10.1038/s41420-024-01857-z (PMC10874379; doi:10.1038/s41420-024-01857-z)

SUPPLEMENTAL MATERIAL

Title: Role of PATJ in Stroke Prognosis by modulating Endothelial to Mesenchymal

1. SUPPLEMENTARY TABLES & FIGURES

Suppl. Table 1: Differentially expressed genes (FDR P-value ≤ 0.05) between CTL and *PATJ* KD cells with Log2 fold-change ≤0.4 (A) and ≥0.4 (B).

SYMBOL	logFC	AveExpr	t	P.Value	adj.P.Val	B	GENENAME
ITM2A	-4,95	9,15	-17,59	0,00	0,00	8,65	integral membrane protein 2A
MGP	-4,20	6,80	-17,22	0,00	0,00	8,53	matrix Gla protein
AK5	-4,08	7,16	-8,46	0,00	0,01	3,67	adenylate kinase 5
WASF3	-4,03	7,10	-10,31	0,00	0,00	5,14	WAS protein family, member 3
MIR99AHG	-3,27	9,29	-13,62	0,00	0,00	7,08	mir-99a-let-7c cluster host gene
FOXG1	-3,13	8,08	-5,80	0,00	0,05	0,86	forkhead box G1
HOXC10	-3,12	5,74	-16,92	0,00	0,00	8,43	homeobox C10
HOXC9	-3,01	6,96	-13,79	0,00	0,00	7,17	homeobox C9
FRMPD4	-3,00	5,15	-21,00	0,00	0,00	9,58	FERM and PDZ domain containing 4
OSBPL6	-2,94	5,90	-14,60	0,00	0,00	7,54	oxysterol binding protein-like 6
RUNX3	-2,92	7,13	-15,25	0,00	0,00	7,81	runt-related transcription factor 3
ROBO1	-2,89	5,72	-15,05	0,00	0,00	7,73	roundabout guidance receptor 1
BMP6	-2,89	7,03	-9,84	0,00	0,00	4,80	bone morphogenetic protein 6
SULT1E1	-2,88	6,00	-14,54	0,00	0,00	7,51	sulfotransferase family 1E member 1
TNIK	-2,81	5,65	-16,43	0,00	0,00	8,26	TRAF2 and NCK interacting kinase
GFRA1	-2,77	7,44	-9,27	0,00	0,00	4,36	GDNF family receptor alpha 1
HOXC8	-2,52	6,74	-12,67	0,00	0,00	6,60	homeobox C8
BICC1	-2,33	6,76	-9,45	0,00	0,00	4,50	BicC family RNA binding protein 1
CA2	-2,31	6,73	-13,52	0,00	0,00	7,03	carbonic anhydrase II
ID1	-2,28	8,53	-8,74	0,00	0,00	3,91	inhibitor of DNA binding 1, dominant negative helix-loop-helix protein
SLITRK4	-2,20	6,50	-9,77	0,00	0,00	4,75	SLIT and NTRK-like family, member 4
PDE10A	-2,12	6,40	-17,50	0,00	0,00	8,63	phosphodiesterase 10A
SYT15	-2,09	7,92	-9,42	0,00	0,00	4,48	synaptotagmin XV
FGL2	-2,08	7,06	-10,59	0,00	0,00	5,33	fibrinogen-like 2
LIMCH1	-2,03	5,68	-15,53	0,00	0,00	7,92	LIM and calponin homology domains 1
TEX15	-1,92	4,90	-15,70	0,00	0,00	7,99	testis expressed 15
FARP1	-1,83	8,26	-8,91	0,00	0,00	4,06	FERM, ARH/RhoGEF and pleckstrin domain protein 1
ZFHX4	-1,78	7,30	-7,70	0,00	0,01	2,96	zinc finger homeobox 4
SOX2	-1,74	6,86	-11,09	0,00	0,00	5,66	SRY box 2
BGN	-1,62	10,76	-18,53	0,00	0,00	8,94	biglycan
SFRP1	-1,61	5,99	-5,60	0,00	0,05	0,62	secreted frizzled-related protein 1
HOXC6	-1,57	6,02	-9,03	0,00	0,00	4,16	homeobox C6
TMOD1	-1,56	7,25	-7,26	0,00	0,01	2,52	tropomodulin 1
PHGDH	-1,51	6,97	-11,97	0,00	0,00	6,20	phosphoglycerate dehydrogenase
SEMA3E	-1,50	4,82	-8,07	0,00	0,01	3,31	sema domain, immunoglobulin domain (Ig), short basic domain, secreted, (semaphorin) 3E
SYT15	-1,41	7,96	-6,97	0,00	0,02	2,22	Homo sapiens synaptotagmin XV (SYT15), transcript variant a, mRNA.
TRO	-1,40	4,30	-8,73	0,00	0,00	3,90	trophinin
KCNIP1	-1,36	5,09	-14,48	0,00	0,00	7,48	Kv channel interacting protein 1
TAF7L	-1,35	5,31	-11,25	0,00	0,00	5,77	TATA box binding protein associated factor 7 like
ADORA1	-1,35	7,31	-7,21	0,00	0,01	2,47	adenosine A1 receptor
PAK3	-1,28	5,45	-6,65	0,00	0,02	1,87	p21 protein (Cdc42/Rac)-activated kinase 3
WNT2B	-1,17	6,91	-6,15	0,00	0,04	1,29	wingless-type MMTV integration site family, member 2B
CGNL1	-1,16	5,21	-8,22	0,00	0,01	3,45	cingulin-like 1
MEIS1	-1,15	7,71	-8,87	0,00	0,00	4,02	Meis homeobox 1
LIMA1	-1,14	10,15	-9,55	0,00	0,00	4,57	LIM domain and actin binding 1
AR	-1,14	6,29	-6,49	0,00	0,03	1,69	androgen receptor
SEMA3D	-1,12	5,31	-5,80	0,00	0,05	0,86	sema domain, immunoglobulin domain (Ig), short basic domain, secreted, (semaphorin) 3D
PCDH1	-1,08	7,50	-9,21	0,00	0,00	4,30	protocadherin 1
RPGR	-1,05	8,98	-8,58	0,00	0,01	3,78	retinitis pigmentosa GTPase regulator
PREX2	-1,05	5,45	-7,85	0,00	0,01	3,11	phosphatidylinositol-3,4,5-trisphosphate-dependent Rac exchange factor 2
PFN2	-1,00	9,76	-5,62	0,00	0,05	0,64	profilin 2
FHOD3	-0,97	6,98	-8,02	0,00	0,01	3,27	formin homology 2 domain containing 3
ANKRD50	-0,97	6,59	-7,59	0,00	0,01	2,85	ankyrin repeat domain 50
TSPAN18	-0,95	6,83	-7,02	0,00	0,02	2,27	tetraspanin 18
DCHS1	-0,95	5,33	-6,77	0,00	0,02	2,00	dachsous cadherin-related 1
CDH3	-0,95	7,57	-6,17	0,00	0,03	1,31	cadherin 3, type 1, P-cadherin (placental)
FHDC1	-0,94	5,36	-6,05	0,00	0,04	1,18	FH2 domain containing 1
ALDH1A1	-0,91	5,14	-7,08	0,00	0,02	2,34	aldehyde dehydrogenase 1 family, member A1
FAM218A	-0,90	5,66	-7,35	0,00	0,01	2,61	family with sequence similarity 218, member A
SLITRK5	-0,89	5,70	-10,11	0,00	0,00	4,99	SLIT and NTRK-like family, member 5
LPAR6	-0,88	9,60	-5,95	0,00	0,04	1,06	lysophosphatidic acid receptor 6
SYNE2	-0,85	8,46	-6,47	0,00	0,03	1,67	spectrin repeat containing, nuclear envelope 2
SCG5	-0,84	6,06	-6,09	0,00	0,04	1,22	secretogranin V
CHD3	-0,83	8,04	-6,45	0,00	0,03	1,65	chromodomain helicase DNA binding protein 3
RP11-380G5.	-0,83	8,81	-5,60	0,00	0,05	0,62	---
ARHGAP6	-0,82	5,88	-7,49	0,00	0,01	2,76	Rho GTPase activating protein 6
CD109	-0,82	7,51	-6,54	0,00	0,03	1,74	CD109 molecule
FAM101B	-0,82	7,37	-6,25	0,00	0,03	1,41	family with sequence similarity 101, member B
QRFPR	-0,82	6,53	-6,08	0,00	0,04	1,21	pyroglutamylated RFamide peptide receptor
PCDH9	-0,79	6,36	-10,55	0,00	0,00	5,31	protocadherin 9
CBLN2	-0,78	5,31	-7,05	0,00	0,02	2,30	cerebellin 2 precursor
ERRFI1	-0,77	10,97	-9,16	0,00	0,00	4,26	ERBB receptor feedback inhibitor 1
GLDC	-0,77	6,83	-8,22	0,00	0,01	3,45	glycine dehydrogenase (decarboxylating)
TMCC3	-0,77	6,10	-7,50	0,00	0,01	2,77	transmembrane and coiled-coil domain family 3
RNF150	-0,75	5,07	-8,49	0,00	0,01	3,70	ring finger protein 150
ZNF814	-0,75	7,89	-6,44	0,00	0,03	1,63	zinc finger protein 814
ZNF718	-0,74	6,97	-7,73	0,00	0,01	2,99	zinc finger protein 718
CXADR	-0,72	6,67	-5,71	0,00	0,05	0,76	coxsackie virus and adenovirus receptor
PTPRM	-0,71	9,97	-6,35	0,00	0,03	1,53	protein tyrosine phosphatase, receptor type, M
CDH4	-0,70	5,51	-6,06	0,00	0,04	1,19	cadherin 4, type 1, R-cadherin (retinal)
MPP6	-0,69	8,60	-5,83	0,00	0,05	0,90	membrane protein, palmitoylated 6
RNASE1	-0,69	5,91	-5,77	0,00	0,05	0,83	ribonuclease, RNase A family, 1 (pancreatic)
TBX18	-0,68	6,15	-7,13	0,00	0,01	2,39	T-box 18
PKIB	-0,67	4,97	-5,74	0,00	0,05	0,80	protein kinase (cAMP-dependent, catalytic) inhibitor beta
NNMT	-0,64	11,34	-7,58	0,00	0,01	2,85	nicotinamide N-methyltransferase
SPARCL1	-0,64	5,02	-5,77	0,00	0,05	0,83	SPARC like 1
FAM195B	-0,60	9,23	-7,13	0,00	0,01	2,38	family with sequence similarity 195, member B
IFNGR1	-0,56	9,14	-7,82	0,00	0,01	3,08	interferon gamma receptor 1
RBMS3	-0,56	10,13	-5,66	0,00	0,05	0,69	RNA binding motif, single stranded interacting protein 3
SEC14L2	-0,54	6,73	-5,58	0,00	0,05	0,59	SEC14-like lipid binding 2
BPGM	-0,53	8,87	-5,72	0,00	0,05	0,76	2,3-bisphosphoglycerate mutase
CCL27	0,45	7,25	6,37	0,00	0,03	1,55	chemokine (C-C motif) ligand 27
MSRA	0,45	8,60	6,35	0,00	0,03	1,52	methionine sulfoxide reductase A
LINC00346	0,54	6,62	5,98	0,00	0,04	1,09	long intergenic non-protein coding RNA 346
OXCT1	0,57	9,63	5,85	0,00	0,05	0,93	3-oxoacid CoA-transferase 1
ACYP2	0,61	9,60	8,21	0,00	0,01	3,45	acylphosphatase 2, muscle type
DUSP16	0,61	6,64	5,77	0,00	0,05	0,82	dual specificity phosphatase 16
C10orf25	0,62	7,45	5,72	0,00	0,05	0,77	chromosome 10 open reading frame 25
AHR	0,65	10,88	7,16	0,00	0,01	2,42	aryl hydrocarbon receptor
QDPR	0,65	7,74	7,14	0,00	0,01	2,39	quinoid dihydropteridine reductase
PTGFR	0,65	5,52	5,73	0,00	0,05	0,78	prostaglandin F receptor (FP)
TRPM2	0,66	7,70	5,78	0,00	0,05	0,85	transient receptor potential cation channel, subfamily M, member 2
LGALSL	0,67	9,12	8,05	0,00	0,01	3,30	lectin, galactoside-binding-like
GABBR1	0,70	8,48	6,46	0,00	0,03	1,65	gamma-aminobutyric acid (GABA) B receptor, 1
ARHGEF35	0,70	8,46	5,75	0,00	0,05	0,81	Rho guanine nucleotide exchange factor 35
TSACC	0,74	7,34	5,72	0,00	0,05	0,76	TSSK6 activating co-chaperone
DUSP16	0,81	8,91	5,66	0,00	0,05	0,69	dual specificity phosphatase 16
DUSP16	0,83	7,62	6,14	0,00	0,04	1,28	dual specificity phosphatase 16
APLF	0,85	7,44	6,77	0,00	0,02	2,00	aprataxin and PNKP like factor
NEU1	0,86	9,51	7,24	0,00	0,01	2,51	sialidase 1 (lysosomal sialidase)
GREM1	0,88	7,32	5,79	0,00	0,05	0,86	gremlin 1, DAN family BMP antagonist [Source:HGNC Symbol;Acc:HGNC:2001]
OCLN	0,92	8,61	8,17	0,00	0,01	3,41	occludin
C7orf31	0,96	7,61	6,20	0,00	0,03	1,35	chromosome 7 open reading frame 31
PDZD2	0,99	8,11	13,59	0,00	0,00	7,07	PDZ domain containing 2
NIPAL2	1,01	7,74	6,10	0,00	0,04	1,23	NIPA-like domain containing 2
SERINC2	1,04	9,22	7,75	0,00	0,01	3,01	serine incorporator 2
PDE4DIP	1,09	7,58	10,06	0,00	0,00	4,96	phosphodiesterase 4D interacting protein
TMEM171	1,21	10,06	6,06	0,00	0,04	1,19	transmembrane protein 171
GPAT2	1,31	7,68	8,85	0,00	0,00	4,01	glycerol-3-phosphate acyltransferase 2, mitochondrial
LURAP1L	1,32	4,91	8,88	0,00	0,00	4,03	leucine rich adaptor protein 1-like
NAP1L5	1,39	7,35	10,46	0,00	0,00	5,24	nucleosome assembly protein 1-like 5
CYP4V2	1,43	5,70	9,35	0,00	0,00	4,42	cytochrome P450, family 4, subfamily V, polypeptide 2
MID2	1,59	6,75	13,80	0,00	0,00	7,17	midline 2
OSBPL1A	1,62	7,15	5,59	0,00	0,05	0,60	oxysterol binding protein-like 1A
ELOVL7	1,84	6,08	8,32	0,00	0,01	3,55	ELOVL fatty acid elongase 7
LIPH	1,99	7,22	18,04	0,00	0,00	8,80	lipase, member H
RASGRP3	2,00	6,37	5,72	0,00	0,05	0,76	RAS guanyl releasing protein 3 (calcium and DAG-regulated)
NOSTRIN	2,02	6,90	10,02	0,00	0,00	4,93	nitric oxide synthase trafficking

Suppl. Table 2. Over-Representation Analysis (ORA) of functional categories with an FDR-cut off of 0.05 obtained using WikiPathways.

	ID	Description	GeneRatio	BgRatio	pvalue	p.adjust	qvalue
WP1984	WP1984	Integrated breast cancer pathway	151/6764	155/7856	1,3267E-06	0,000388256	0,00028765
WP3888	WP3888	VEGFA-VEGFR2 signaling pathway	409/6764	439/7856	1,35281E-06	0,000388256	0,00028765
WP437	WP437	EGF/EGFR signaling pathway	159/6764	164/7856	2,30651E-06	0,000441313	0,000326958
WP366	WP366	TGF-beta signaling pathway	130/6764	133/7856	3,7121E-06	0,000532687	0,000394655
WP3932	WP3932	Focal adhesion: PI3K-Akt-mTOR-signaling pathway	290/6764	309/7856	8,36393E-06	0,000960179	0,000711374
WP5124	WP5124	Alzheimer's disease	254/6764	270/7856	1,7966E-05	0,001489378	0,001103445
WP382	WP382	MAPK signaling pathway	235/6764	249/7856	1,81631E-05	0,001489378	0,001103445
WP236	WP236	Adipogenesis	127/6764	131/7856	2,67026E-05	0,00170503	0,001263217
WP306	WP306	Focal adhesion	192/6764	202/7856	2,67339E-05	0,00170503	0,001263217
WP2064	WP2064	Neural crest differentiation	100/6764	102/7856	3,28646E-05	0,001886427	0,00139761
WP4806	WP4806	EGFR tyrosine kinase inhibitor resistance	83/6764	84/7856	4,75356E-05	0,002480496	0,001837742
WP481	WP481	Insulin signaling	154/6764	161/7856	5,71858E-05	0,002735386	0,002026584
WP45	WP45	G1 to S cell cycle control	64/6764	64/7856	6,63801E-05	0,002930937	0,002171463
WP179	WP179	Cell cycle	118/6764	122/7856	7,96663E-05	0,003169756	0,002348398
WP2853	WP2853	Endoderm differentiation	140/6764	146/7856	8,28333E-05	0,003169756	0,002348398
WP231	WP231	TNF-alpha signaling pathway	92/6764	94/7856	9,46153E-05	0,003394324	0,002514775
WP4787	WP4787	Osteoblast differentiation and related diseases	116/6764	120/7856	0,000101286	0,003419893	0,002533719
WP4754	WP4754	IL-18 signaling pathway	260/6764	279/7856	0,000119551	0,003705214	0,002745106
WP4239	WP4239	Epithelial to mesenchymal transition in colorectal cancer	157/6764	165/7856	0,000125627	0,003705214	0,002745106
WP4172	WP4172	PI3K-Akt signaling pathway	319/6764	345/7856	0,000129102	0,003705214	0,002745106
WP2037	WP2037	Prolactin signaling pathway	75/6764	76/7856	0,000145173	0,003968075	0,002939854
WP51	WP51	Regulation of actin cytoskeleton	144/6764	151/7856	0,000169151	0,00441331	0,003269718
WP4659	WP4659	Gastrin signaling pathway	110/6764	114/7856	0,000206773	0,004955879	0,003671695
WP2446	WP2446	Retinoblastoma gene in cancer	86/6764	88/7856	0,000207214	0,004955879	0,003671695
WP5087	WP5087	Malignant pleural mesothelioma	393/6764	429/7856	0,000223395	0,005108703	0,003784919
WP3584	WP3584	MECP2 and associated Rett syndrome	71/6764	72/7856	0,0002527	0,005108703	0,003784919
WP4255	WP4255	Non-small cell lung cancer	71/6764	72/7856	0,0002527	0,005108703	0,003784919
WP2018	WP2018	RANKL/RANK signaling pathway	55/6764	55/7856	0,000258105	0,005108703	0,003784919
WP2272	WP2272	Pathogenic Escherichia coli infection	55/6764	55/7856	0,000258105	0,005108703	0,003784919
WP23	WP23	B cell receptor signaling pathway	95/6764	98/7856	0,000313492	0,005998154	0,004443892
WP2380	WP2380	Brain-derived neurotrophic factor (BDNF) signaling pathway	137/6764	144/7856	0,000354994	0,006573113	0,004869866
WP3303	WP3303	RAC1/PAK1/p38/MMP2 pathway	68/6764	69/7856	0,000382215	0,006678091	0,004947641
WP4666	WP4666	Hepatitis B infection	146/6764	154/7856	0,000390889	0,006678091	0,004947641
WP2261	WP2261	Glioblastoma signaling pathways	81/6764	83/7856	0,000395566	0,006678091	0,004947641
WP4685	WP4685	Melanoma	67/6764	68/7856	0,000438571	0,007192558	0,005328797
WP4262	WP4262	Breast cancer pathway	148/6764	157/7856	0,000828657	0,01321247	0,009788809
WP4155	WP4155	Endometrial cancer	62/6764	63/7856	0,000869629	0,013491001	0,009995167
WP4263	WP4263	Pancreatic adenocarcinoma pathway	86/6764	89/7856	0,000931704	0,014073628	0,010426821
WP2034	WP2034	Leptin signaling pathway	74/6764	76/7856	0,000966759	0,014228715	0,010541722
WP5072	WP5072	Modulators of TCR signaling and T cell activation	60/6764	61/7856	0,001141749	0,016384098	0,012138594
WP4217	WP4217	Ebola virus pathway in host	125/6764	132/7856	0,001217583	0,016613469	0,01230853
WP4946	WP4946	DNA repair pathways, full network	115/6764	121/7856	0,001242312	0,016613469	0,01230853
WP4216	WP4216	Chromosomal and microsatellite instability in colorectal cancer	72/6764	74/7856	0,001244563	0,016613469	0,01230853
WP5053	WP5053	Development of ureteric collection system	59/6764	60/7856	0,001307773	0,016614613	0,012309378
WP4658	WP4658	Small cell lung cancer	94/6764	98/7856	0,001312516	0,016614613	0,012309378
WP254	WP254	Apoptosis	83/6764	86/7856	0,001331485	0,016614613	0,012309378
WP4352	WP4352	Ciliary landscape	204/6764	220/7856	0,001439293	0,017577749	0,013022943
WP4321	WP4321	Thermogenesis	103/6764	108/7856	0,001527809	0,017903461	0,013264255
WP3931	WP3931	Embryonic stem cell pluripotency pathways	113/6764	119/7856	0,001528344	0,017903461	0,013264255
WP4396	WP4396	Nonalcoholic fatty liver disease	150/6764	160/7856	0,001630571	0,018718953	0,013868434
WP2036	WP2036	TNF-related weak inducer of apoptosis (TWEAK) signaling pathway	42/6764	42/7856	0,001829682	0,02019687	0,014963388
WP466	WP466	DNA replication	42/6764	42/7856	0,001829682	0,02019687	0,014963388
WP2359	WP2359	Parkin-ubiquitin proteasomal system pathway	68/6764	70/7856	0,00205433	0,022248781	0,016483601
WP2355	WP2355	Corticotropin-releasing hormone signaling pathway	89/6764	93/7856	0,002294393	0,024388544	0,018068901
WP3937	WP3937	Microglia pathogen phagocytosis pathway	40/6764	40/7856	0,002472283	0,025801648	0,019115837
WP395	WP395	IL-4 signaling pathway	54/6764	55/7856	0,002568358	0,025894157	0,019184375
WP5115	WP5115	Network map of SARS-CoV-2 signaling pathway	205/6764	222/7856	0,002571371	0,025894157	0,019184375
WP138	WP138	Androgen receptor signaling pathway	87/6764	91/7856	0,00286048	0,027829079	0,020617913
WP69	WP69	T-cell receptor (TCR) signaling pathway	87/6764	91/7856	0,00286048	0,027829079	0,020617913
WP411	WP411	mRNA processing	125/6764	133/7856	0,003040461	0,029087079	0,021549936
WP536	WP536	Calcium regulation in cardiac cells	142/6764	152/7856	0,003334311	0,031375323	0,023245242
WP4656	WP4656	Joubert syndrome	74/6764	77/7856	0,003806053	0,035236687	0,026106036
WP3614	WP3614	Photodynamic therapy-induced HIF-1 survival signaling	37/6764	37/7856	0,003882529	0,035374149	0,026207879
WP4018	WP4018	Clear cell renal cell carcinoma pathways	83/6764	87/7856	0,004422628	0,039665442	0,029387197
WP289	WP289	Myometrial relaxation and contraction pathways	147/6764	158/7856	0,004598611	0,040331041	0,029880324
WP615	WP615	Senescence and autophagy in cancer	102/6764	108/7856	0,004637367	0,040331041	0,029880324
WP61	WP61	Notch signaling pathway (Netpath)	61/6764	63/7856	0,004867962	0,041704631	0,030897985
WP5094	WP5094	Orexin receptor pathway	144/6764	155/7856	0,005905013	0,04947375	0,036653943
WP4746	WP4746	Thyroid hormones production and peripheral downstream signaling effects	90/6764	95/7856	0,005947193	0,04947375	0,036653943

Suppl. Table 3. Most significant pathways (Adjusted P-val <0,05) modulated due to *PATJ* KD identified through Reactome.

	Total	Upregulated	Downregulated		
Pathway	modulated genes	Genes	Genes2	Significance	p-value
Ectoderm differentiation	9	CCL2,TRPM2,EDA2R	FZD4,GRAMD1B,ZFHX4,PTPRB,SKIL,SOX2	3,15	0,000716
G protein signaling pathways	7	PRKAR1B,PDE4A,PDE1B,AKAP3,PRKD1	PDE4B,GNG4	3,02	0,000959
Photodynamic therapy-induced unfolded protein response	4	DDIT3,NARS,ATF3	SULT1E1	2,98	0,001041
Ras signaling	10	PLCG2,RASGRP3,KSR2	KDR,RASGRF1,GNG4,RASSF5,PAK3,ETS2,PLCE1	2,92	0,001209
Transcriptional cascade regulating adipogenesis	3	DDIT3	GATA2,KLF5	2,92	0,001213
Myometrial relaxation and contraction pathways	9	PLCG2,PRKD1,PRKAR1B,ATF3	PKIB,RGS9,ETS2,PDE4B,GNG4	2,86	0,001392
Epithelial to mesenchymal transition in colorectal cancer	9	OCLN	NRP2,CDKL2,ID1,CLDN5,COL4A5,COL4A6,WNT2B,FZD4	2,76	0,001731
TGF-beta signaling pathway	8	NEDD4L,STAMBPL1,ATF3	TERT,SKIL,MEF2C,SIK1,ITGA2	2,67	0,002144
Pre-implantation embryo	5	DDIT3,BATF3	NR3C2,GATA2,SOX2	2,49	0,003201
VEGFA-VEGFR2 signaling pathway	16	PRKD1,CCL2,OCLN,KATNAL2,PDE4DIP	NRP2,MEF2C,S1PR1,TAL1,GATA2,TRAF3IP2,HTRA1,KANK1,CGNL1,MICAL2,TMOD1	2,38	0,004168
Lung fibrosis	5	CCL2,GREM1	SKIL,EDN1,TERT	2,34	0,004553
Hair follicle development: cytodifferentiation - part 3 of 3	6	PERP,FST	SFRP1,SOX2,RUNX3,BMP6	2,32	0,004747
Hippo-Merlin signaling dysregulation	8	PRKAR1B	CDH3,CDH4,ITGA2,ITGB8,KDR,PAK3,PPP1R14A	2,28	0,005271
Hfe effect on hepcidin production	2		BMP6,ID1	2,25	0,005673
Sudden infant death syndrome (SIDS) susceptibility pathways	8	PRKAR1B,MYB	HES1,MEF2C,RUNX3,GATA2,AR,SOX2	2,22	0,006016
Glucocorticoid receptor pathway	5	CCL2,PRRG4	PDE4B,PLK2,ALOX5AP	2,15	0,007066
Exercise-induced circadian regulation	4	HLA-DMA	GSTM3,CLDN5,GFRA1	2,06	0,008745
Folate-alcohol and cancer pathway hypotheses	2		ALDH1A1,CBS	2,02	0,00951
Phosphodiesterases in neuronal function	4	PDE1B,PDE4A	PDE10A,PDE4B	1,91	0,012324
Burn wound healing	6	CCL2,FST,CD248,IL15	KDR,PECAM1	1,87	0,013594
TGF-beta receptor signaling	4	FST	SMAD6,SKIL,RUNX3	1,85	0,013983
Mesodermal commitment pathway	7	EXT2	ZFHX4,KLF5,MEIS1,SMAD6,SOX2,FZD4	1,79	0,016084
Clear cell renal cell carcinoma pathways	5	BHLHE41	KCNJ2,PTEN,PHGDH,KDR	1,79	0,016135
White fat cell differentiation	3	DDIT3	KLF5,GATA2	1,78	0,016597
TGF-beta receptor signaling in skeletal dysplasias	4	FST	SMAD6,SKIL,RUNX3	1,75	0,017709
Purinergic signaling	3	P2RX4	ADORA1,LPAR6	1,74	0,018033
Development of ureteric collection system	4	FST,GREM1	ROBO1,GFRA1	1,7	0,019782
PodNet: protein-protein interactions in the podocyte	11	OCLN	SMAD6,EFNB1,SULF1,ROBO1,CDH3,PLCE1,ITGA2,KDR,KHDRBS3,COL4A5	1,7	0,020101
ATF4 activates genes in response to endoplasmic reticulum stress	3	DDIT3,CCL2,ATF3		1,61	0,024445
Olfactory receptor activity	1	OR2B6		1,61	0,024551
Endochondral ossification with skeletal dysplasias	4		BMP6,MEF2C,RUNX3,MGP	1,59	0,025599
Endochondral ossification	4		BMP6,MEF2C,RUNX3,MGP	1,59	0,025599
GPR40 pathway	2	PLCG2	PLCE1	1,59	0,025946
Melanoma	4	MITF	KDR,PREX2,PTEN	1,53	0,029533
miRNA in muscle cell differentiation	3	PRKAR1B,PRKD1	MEF2C	1,5	0,031929
Estrogen metabolism	2	ARSD	SULT1E1	1,44	0,036572
Sulfation biotransformation reaction	2	SULT1C2	SULT1E1	1,44	0,036572
Mammary gland development pathway - Embryonic development (Stage 1 of 4)	2		SFRP1,TERT	1,44	0,036572
MECP2 and associated Rett syndrome	4	FGF5	MEF2C,FOXG1,PTEN	1,43	0,036843
Vitamin D receptor pathway	7	SULT1C2	CST6,CLMN,CBS,ID1,SFRP1,BMP6	1,4	0,039435
Prostaglandin synthesis and regulation	3	MITF	EDN1,PTGFRN	1,37	0,042771
Embryonic stem cell pluripotency pathways	5	FGF5	FZD4,PTEN,SMAD6,WNT2B	1,32	0,047442
Phosphoinositides metabolism	3	PLCG2	PTEN,PLCE1	1,3	0,050048
PI3K-Akt signaling pathway	11	IL7R,FGF5,MYB	COL4A5,ITGA2,COL4A6,PTEN,ITGB8,KDR,LPAR6,GNG4	1,29	0,050848
Focal adhesion	7		ITGB8,KDR,ITGA2,PAK3,PTEN,COL4A6,RASGRF1	1,28	0,052052
Deubiquitination	2	STAMBPL1	PTEN	1,27	0,053199
GDNF/RET signaling axis	2	GREM1	GFRA1	1,24	0,05724
Extracellular vesicles in the crosstalk of cardiac cells	2		PTEN,ETS2	1,24	0,05724

Suppl. Fig. 1. (A) *PATJ* KD clones generated by infection with lentiviral particles harboring *PATJ*-shRNA were selected for the 80 kDa PATJ specie. **(B)** Clones 1, 2 and 3 were selected for further analysis and cell viability and proliferation was assessed with an MTT assay. No significant differences were observed between CTL and *PATJ* KD cells after 24 hr growth.

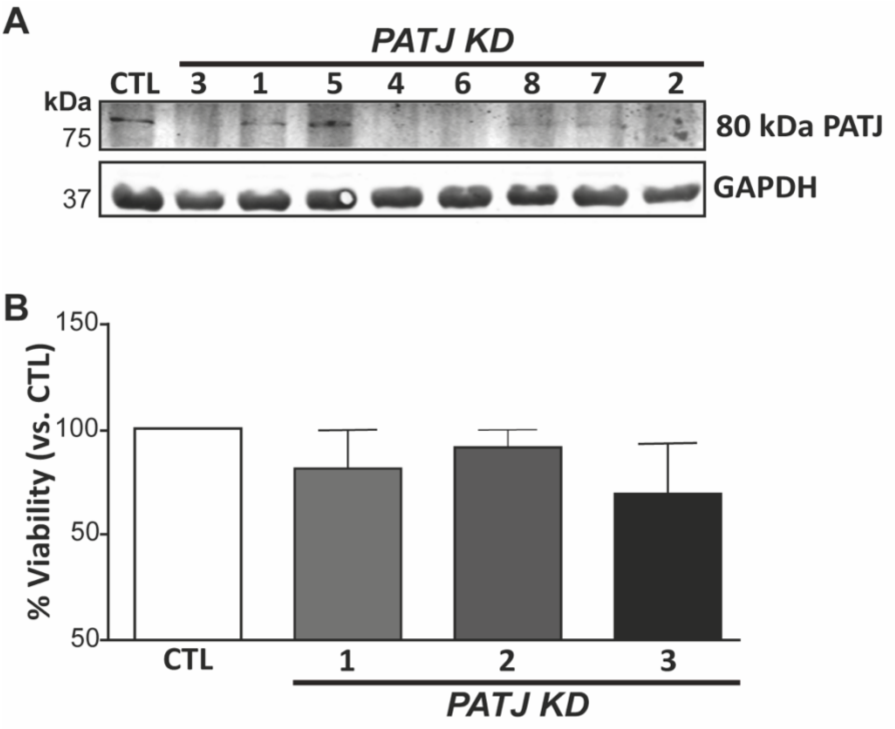

Suppl. Fig. 2. Summary of Affymetrix Expression Array Results comparing hCMEC/D3 *PATJ* KD with CTL cells

KD PATJ vs Control
- KD PATJ: 4 samples, Control: 4 samples

Filter criteria:
- Fold Change: > 2 or < -2
- P-val: < 0.05

Total number of genes: 21448
- Genes passed filter criteria: 341 (1.59%)
  - Up-Regulated: 137 (40.18%)
  - Down-Regulated: 204 (59.82%)

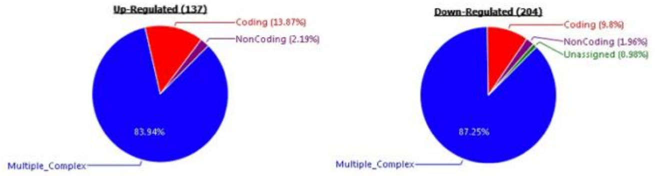

Group	Total	Passed Filter	Up-Regulated	Down-Regulated
Multiple_Complex	16081	293	115	178
Coding	3878	39	19	20
NonCoding	1116	7	3	4
Unassigned	352	2	0	2
Pseudogene	21	0	0	0

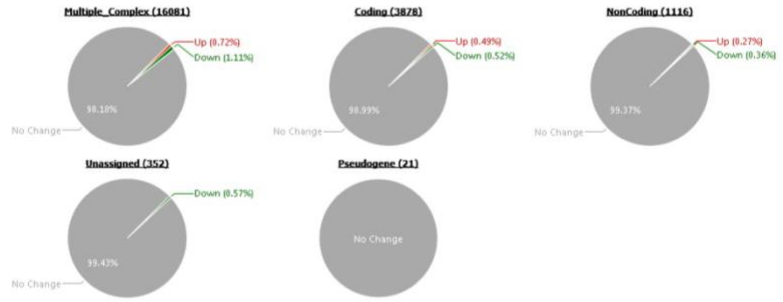

Suppl. Fig.3: Principal Component Analysis of the Affymetrix GeneChip Array Clarion S comparing hCMEC/D3 *PATJ* KD vs CTL cells.

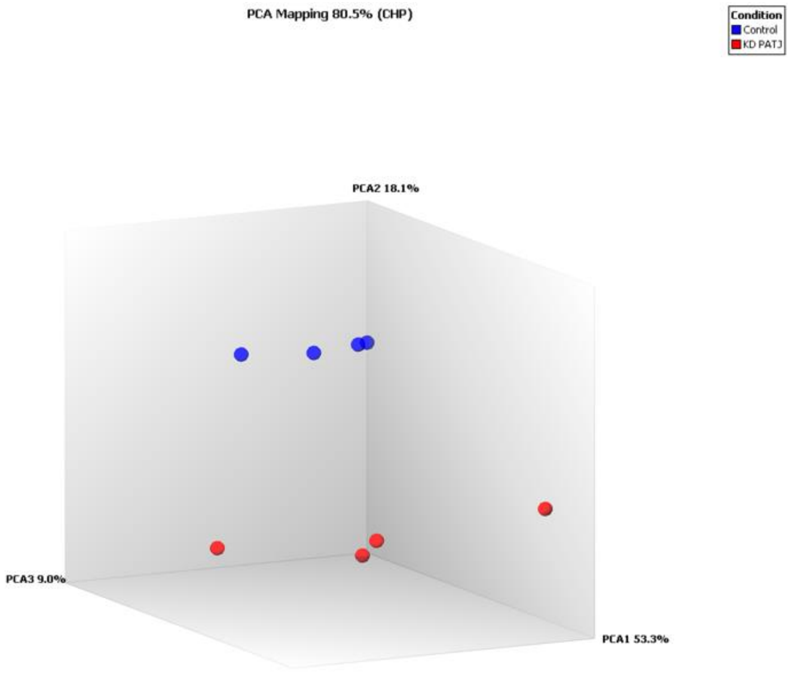

Supplement: Supplementary file 2 — Supplementary Material [file 41420_2024_1857_MOESM2_ESM.pdf]
